# Physiological and comparative transcriptome analyses reveal the mechanisms underlying waterlogging tolerance in a rapeseed *anthocyanin-more* mutant

**DOI:** 10.1186/s13068-022-02155-5

**Published:** 2022-05-20

**Authors:** Li-Na Ding, Rui Liu, Teng Li, Ming Li, Xiao-Yan Liu, Wei-Jie Wang, Yan-Kun Yu, Jun Cao, Xiao-Li Tan

**Affiliations:** School of Life Sciences, Jiangsu University, Zhenjiang, China

**Keywords:** *Brassica napus*, Waterlogging stress, Anthocyanin, Transcriptome, Physiological, Candidate gene

## Abstract

**Background:**

Rapeseed (*Brassica napus*) is the second largest oil crop worldwide. It is widely used in food, energy production and the chemical industry, as well as being an ornamental. Consequently, it has a large economic value and developmental potential. Waterlogging is an important abiotic stress that restricts plant growth and development. However, little is known about the molecular mechanisms underlying waterlogging tolerance in *B. napus.*

**Results:**

In the present study, the physiological changes and transcriptomes of germination-stage rapeseed in response to waterlogging stress were investigated in the *B. napus* cultivar ‘Zhongshuang 11’ (ZS11) and its *anthocyanin-more* (*am*) mutant, which was identified in our previous study. The mutant showed stronger waterlogging tolerance compared with ZS11, and waterlogging stress significantly increased anthocyanin, soluble sugar and malondialdehyde contents and decreased chlorophyll contents in the mutant after 12 days of waterlogging. An RNA-seq analysis identified 1370 and 2336 differently expressed genes (DEGs) responding to waterlogging stress in ZS11 and *am*, respectively. An enrichment analysis revealed that the DEGs in ZS11 were predominately involved in carbohydrate metabolism, whereas those in the *am* mutant were particularly enriched in plant hormone signal transduction and response to endogenous stimulation. In total, 299 DEGs were identified as anthocyanin biosynthesis-related structural genes (24) and regulatory genes encoding transcription factors (275), which may explain the increased anthocyanin content in the *am* mutant. A total of 110 genes clustered in the plant hormone signal transduction pathway were also identified as DEGs, including 70 involved in auxin and ethylene signal transduction that were significantly changed in the mutant. Furthermore, the expression levels of 16 DEGs with putative roles in anthocyanin accumulation and biotic/abiotic stress responses were validated by quantitative real-time PCR as being consistent with the transcriptome profiles.

**Conclusion:**

This study provides new insights into the molecular mechanisms of increased anthocyanin contents in rapeseed in response to waterlogging stress, which should be useful for reducing the damage caused by waterlogging stress and for further breeding new rapeseed varieties with high waterlogging tolerance.

**Supplementary Information:**

The online version contains supplementary material available at 10.1186/s13068-022-02155-5.

## Background

Rapeseed (*Brassica napus*) is a main oil crop worldwide and has the largest cultivated area in China. Rapeseed oil contains high oleic acid, low saturated fatty acid, moderate linoleic acid, linolenic acid and many vitamins and sterols, which are important nutrient resources in edible vegetable oil [1, 2]. In addition, the applications of rapeseed oil in chemical, pharmaceutical and bioenergy fields are increasing [3, 4].

During growth and development, rapeseed encounters various adverse stresses, such as low temperature, salt, drought, waterlogging, heavy metal ions and pathogens, which can hinder the growth and reduce the yield by affecting gene expression and cell physiological metabolism. Among these stresses, rapeseed is especially sensitive to waterlogging stress owing to the lack of aerenchymal tissue [5]. Similar to wheat and corn, waterlogging stress also results in decreases in the photosynthetic rate, chlorophyll content, root absorption function and antioxidant enzyme activity of rapeseed, hindering the development of pods and eventually leading to yield reductions [6–11]. The Yangtze River Basin is the main rapeseed planting area in China, and heavy rainfalls during the growing season in this area increase the soil moisture and humidity, which also accelerates the propagation and spread of *Sclerotinia sclerotiorum* and other pathogens [8, 12]. Although measures adopted in production, such as ditching and drainage, plowing the land and increasing organic fertilizer, alleviate the harm to rapeseed caused by waterlogging stress to a certain extent, they undoubtedly increase the labor costs [13]. Therefore, breeding new rapeseed varieties with high waterlogging tolerance is still the most economical and effective way to reduce the damage, and in-depth analyses of waterlogging-tolerance mechanism are of great significance to achieve this breeding goal.

During waterlogging stress, plants usually form aerenchyma and radial oxygen loss barriers in adventitious roots and lateral roots to increase the oxygen transport capacity [14–16] or inhibit root extension to reduce energy consumption under hypoxic conditions [17]. However, owing to the lack of aerenchyma and the sensitivity to water, rapeseed can form numerous adventitious roots to maintain a high energy reserve, which improves its adaptability under stress conditions [5, 18]. In addition to morphological changes, plants also induce some positive physiological and molecular responses to waterlogging stress [19]. For example, many metabolic pathways, including glycolytic, fermentation and respiratory metabolic pathways, are changed accordingly. Moreover, some endogenous plant hormones, such as abscisic acid (ABA), ethylene (ET), auxin and brassinolide (BR) are very sensitive to waterlogging stress and can effectively mitigate the adverse effects caused by waterlogging [8, 20–24]. Waterlogging-tolerant rapeseed varieties can also significantly increase the contents of osmoregulation organic matter, such as soluble sugar, soluble protein and proline [7, 25]. The application of nitrogen can also effectively improve the physiological indexes of rapeseed, such as root development, chlorophyll content and antioxidant enzyme activity levels, and reduce the degree of membrane lipid peroxidation to alleviate the damage caused by waterlogging stress [11].

Anthocyanins, as one of the most important water-soluble pigments in plants, have a wide range of biological functions, such as plant abiotic stress adaptation, defense against pathogen invasion, antioxidation and other health-related functions [26–28]. Plant anthocyanin production can be affected by environmental factors, plant hormones, miRNAs (such as miR156, miR165/166 and miR778) and nutritional conditions [29–33]. In the natural state, there are few free anthocyanins, they are predominantly in the form of glycosides combined by glycosylation [26, 34]. Anthocyanins are flavonoids, and their biosynthesis and accumulation are parts of the flavonoid biosynthetic pathway, which is mainly controlled by structural and regulatory genes [35, 36]. Structural genes encode various enzymes involved in anthocyanin synthesis, such as chalcone synthase (CHS), dihydroflavonol 4-reductase (DFR) and anthocyanidin synthase (ANS) [37], whereas regulatory genes are related to plant color and regulate the expression levels of structural genes through a conserved MBW (MYB-bHLH-WD40) complex, which is composed of R2R3-MYB transcription factors (TFs) PAP1/MYB75, PAP2/MYB90, MYB113 and MYB114, basic helix–loop–helix (bHLH) TFs TT8, GL3 and EGL3, and the WD40-repeat protein TTG1 in *Arabidopsis* [38–40]. Similar MBW complexes have been identified in different plant species, such as R2R3-MYB TFs MYB6, ROS1 and SlAN2-like [41–44], and bHLH TFs, such as PPLS1 and bHLH33, which positively regulate anthocyanin biosynthesis [45, 46], whereas MYBL2, MYBC2L2 and SlMYBATV serve as transcriptional repressors of anthocyanin biosynthesis [31, 43, 47]. Owing to the lack of anthocyanin-related mutants, structural and regulatory genes that are important in controlling anthocyanin production in *B. napus* are still not well understood and need further identification and study.

In our previous study, we identified an *anthocyanin-more* (*am*) mutant of a *B. napus* cultivar that produced more anthocyanins compared with the wild type (WT) during growth and development [48]. The *am* mutant was more tolerant to waterlogging stress during *B. napus* seed germination and seedling growth and developmental stages. To systematically analyze the genetic and biochemical bases for the different anthocyanin production capabilities between the *am* mutant and WT, and the molecular mechanisms of the different waterlogging responses with respect to the accumulation of anthocyanin, an RNA sequencing (RNA-seq) analysis of leaf tissues was carried out to analyze transcriptional responses to waterlogging stress over 24 h. This study will expand our understanding of the transcriptional regulation of anthocyanin production and the molecular mechanisms of anthocyanin-induced waterlogging resistance in a rapeseed *am* mutant, and this lays the foundation for further candidate gene identification and the molecular breeding of waterlogging-tolerant rapeseed.

## Results

### Differences in seed germination and seedling growth between the WT and *am* mutant under waterlogging-stress conditions

**Fig. 1:**
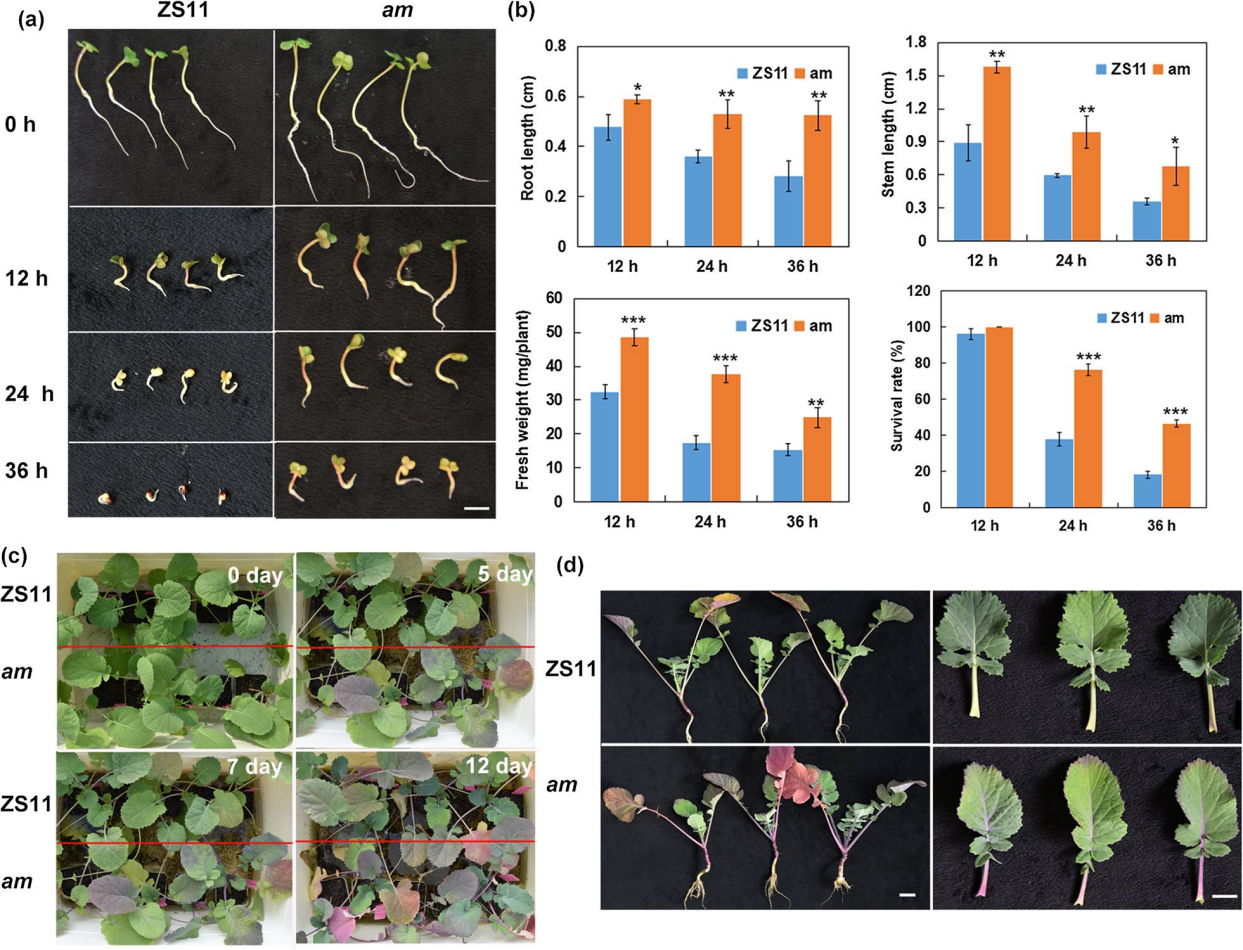
Phenotypic differences between the wild type (WT) and *am* mutant at different periods after waterlogging. **a** The seeds germinated for 36 h were waterlogged for 0, 12, 24 and 36 h. **b** Effects of waterlogging on root lengths, stem lengths and fresh weights of the WT and *am* mutant. Error bars represent the SDs from three replicated experiments. Significant differences between the *am* mutant and WT at different time points are indicated (Student’s *t*-test) as follows: ***P < 0.001; **P < 0.01; *P < 0.05. **c** The young seedlings of the WT ZS11 and *am* mutant were waterlogged for 5, 7 and 12 d. **d** The morphological changes in the ZS11 and *am* mutant seedlings after waterlogging for 12 d. Bars = 1.0 cm

The *am* mutant is a natural mutant of *B. napus* ‘Zhongshuang 11’ (ZS11) identified in our previous study. Compared with WT, the mutant presents purple tissues during different growth and developmental stages of plant [48]. During the early germination (36 h) stages, the root length, stem length and fresh weight of the mutant were significantly higher than those of ZS11 after various time periods (12, 24 and 36 h) under waterlogging stress (Fig. 1a, b). Following an 8-day recovery period, the seedlings rate, growth quantity and growth vigor of the *am* mutant were all superior to those of the WT under the same waterlogging treatment conditions (Additional file 1: Fig. S1). Specifically, no obvious differences in the relative root lengths and fresh weights were found between the two materials after 12 h of waterlogging. After 24 h of treatment and 8 days of recovery, the relative seedling rates of the two materials decreased, but the decline range of *am* was less than that of ZS11. The relative vigor indexes of the two materials also showed significant differences, with ZS11 being only 0.18, whereas that of the *am* mutant was 0.65, indicating a greater tolerance to waterlogging compared with WT. After a 36-h treatment and recovery period, most ZS11 seedlings did not grow normally, and cotyledons could not completely break away from the husks and extend outwards, whereas 46.1% of the *am* mutant germinated and grew normally after waterlogging (Table 1, Additional file 1: Fig. S1). Moreover, the mutant was prone to produce anthocyanins during germination under waterlogging stress, and the stems, petioles and veins appeared purple, whereas the WT plant showed no obvious changes (Fig. 1).

**Table 1:** Effects of waterlogging stress on the seed germination characteristics of ZS11 and the *am* mutant

Waterlogging time (h)	Material	Relative survival rate (%)^a^	Relative root length (%)^b^	Relative stem length (%)^c^	Relative fresh weight (%)^d^	Relative vigor index^e^
12	ZS11	95.8	53.1	62.2	79.0	0.61
	*am*	100	55.1	88.9*	84.5	0.89
24	ZS11	36.8	37.0	47.6	58.8	0.18
	*am*	75.7***	52.6*	86.1**	82.7**	0.65***
36	ZS11	16.7	9.2	34.1	43.4	0.06
	*am*	46.1***	44.2**	76.4**	76.8***	0.35**

^a^Relative survival rate (%) = (survival rate of treated seedlings/survival rate of control seedlings) × 100

^b^Relative root length (%) = (root length of treated seedlings/root length of control seedlings) × 100

^c^Relative stem length (%) = (stem length of treated seedlings/stem length of control seedlings) × 100

^d^Relative fresh weight (%) = (fresh weight of treated seedlings/fresh weight of control seedlings) × 100

^e^Relative vigor index = (survival rate of treated seedlings × stem length of treated seedlings)/(survival rate of control seedlings × stem length of control seedlings)

Statistically significant difference from ZS11 at each time point is indicated (Student’s *t*-test) as follows: ***P < 0.001; **P < 0.01; *P < 0.05

At the seedling stage after waterlogging stress, the *am* mutant had purple stems and leaves prior to the WT, and to a certain extent, as the waterlogging time increased, the purple in these parts gradually deepened (Fig. 1b). When the waterlogging time reached 12 days, the mutant *am* seedlings showed purple-colored veins, petioles and leaf edge areas, and they had well developed root systems, compared with WT seedlings (Fig. 1c). Thus, the *am* mutants were more likely to produce anthocyanins under waterlogging-stress conditions during both the germination and seedling stages, and this may be related to waterlogging tolerance.

### Physiochemical changes in WT and the *am* mutant after waterlogging treatments

**Fig. 2:**
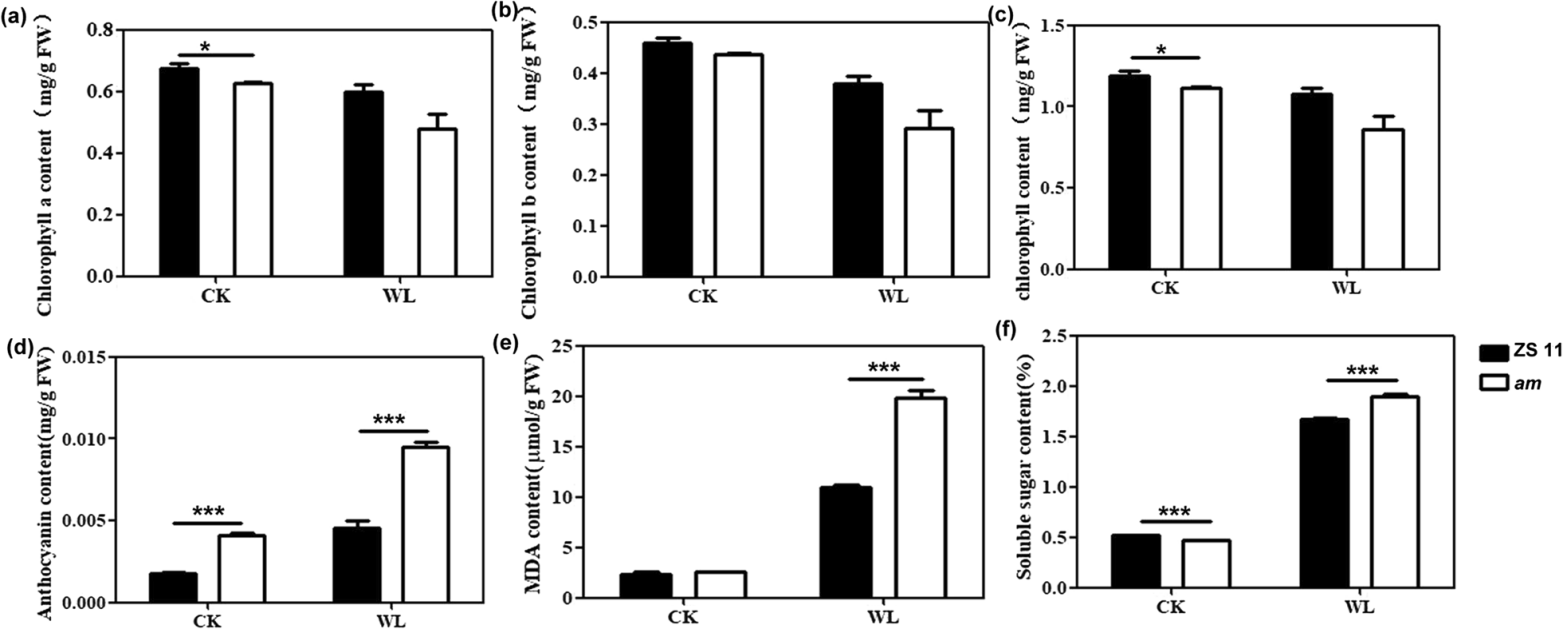
Waterlogging stress had different effects on the physiological indicators of WT and the *am* mutant. **a**–**f** The chlorophyll a (**a**), chlorophyll b (**b**), total chlorophyll (**c**), anthocyanin (**d**), MDA (**e**) and soluble sugar (**f**) contents were determined in the WT and *am* mutant after 12 d of waterlogging stress. Error bars represent the SDs from three replicated experiments. Significant differences between the *am* mutant and WT with and without (CK) waterlogging stress are indicated (Student’s *t*-test) as follows: ***P < 0.001; **P < 0.01; *P < 0.05

Under normal growth conditions, the chlorophyll a, chlorophyll b and total chlorophyll contents of the *am* seedlings leaves were lower than those of the WT. After 12 days of waterlogging stress, the contents of chlorophyll a, chlorophyll b and total chlorophyll in the leaves of WT seedlings decreased by 7.46%, 7.82% and 11.55%, respectively. The changes were more marked in the mutant, with the chlorophyll a, chlorophyll b and total chlorophyll contents decreasing by 14.55%, 14.52% and 25.31%, respectively, which was approximately twice as much as in WT seedlings (Fig. 2a–c). Anthocyanins play important roles in alleviating plant damage caused by environmental stresses. Under both normal water supply and waterlogging-stress conditions, the anthocyanin contents in leaves of the *am* mutant were greater than in WT. Accumulations of anthocyanin in both materials increased by different degrees after waterlogging. However, the amplitude of the anthocyanin increase in *am* seedlings was greater than that of WT seedlings, being fivefold in the mutant and twofold in the WT (Fig. 2d).

Plants trigger or intensify membrane lipid peroxidation in response to environmental stresses. Malondialdehyde (MDA) is one of the products, and it is used as an indicator of lipid peroxidation, indicating the degree of cell membrane peroxidation and the intensity of plant responses to stress [49]. As shown in Fig. 2e, the MDA contents in seedling leaves remained stable in non-waterlogged rapeseed plants, and there was no significant difference between the WT and the mutant. Waterlogging stress treatments triggered a marked increase in the MDA content, which increased by 4.5-fold in WT and eightfold in the mutant. Furthermore, when a plant encounters adversity, soluble sugars accumulate in vivo to regulate the osmotic capacity of the plant and improve its adaptability to stress. After 12 days of waterlogging stress, the soluble sugar levels in seedling leaves of the mutant increased more significantly than in the WT (Fig. 2f). These results indicated that the physiological metabolism of rapeseed seedlings was seriously affected by waterlogging stress, and the mutants may alleviate the corresponding damage by regulating the chlorophyll, anthocyanins, MDA and osmotic adjustment substance levels.

### Transcriptome analysis of WT and the *am* mutant in response to waterlogging stress as assessed by RNA-seq

To further understand the molecular mechanisms underlying the different responses of the two materials to waterlogging stress, transcriptomes of seeding leaves of WT ZS11 and the *am* mutant after a 24-h waterlogging period followed by 8 days of recovery growth were investigated. Corresponding seedlings without waterlogging at the germination stage were used for controls in the screening of differentially expressed genes (DEGs) by RNA-seq. After data quality controls and data filtering, there were 26,187,420 (ZS11CK), 26,777,456 (ZS11WL), 27,536,936 (*am*CK) and 22,117,491 (*am*WL) 150-bp paired-end reads, with an average of 25,654,825 reads per sample. The average clean base reached 7.69 GB per sample, and approximately 83% of the reads from the cDNA libraries were mapped to the *B. napus* reference genome (Table 2). The Q30 percentage was greater than 95%, and the randomness of the sample sequencing was good, which ensures the sequencing quality (Additional file 1: Fig. S2). With an increase in sequencing quantity (number of reads), the number of genes detected also increases. We found that the numbers of genes detected in the treatment and control groups of the two varieties were close to saturation, within a certain Reads Per Kilo bases per Million reads (RPKM) interval, indicating that the sequencing quantity met the requirements (Additional file 1: Fig. S3). A correlation analysis showed that the correlation coefficients of gene expression levels between control and treatment groups of each material were close to 1, whereas the gene expression patterns between WT and mutant samples were different, suggesting that the reproducibility among samples was high and that sequencing data were reliable for further analyses (Additional file 1: Fig. S4).

**Table 2:** Statistics for sequencing reads after filtering and genomes alignment

Sample	Clean reads	Clean base	GC (%)	Q30 (%)	Mapped reads	Unique match
ZS11CK	26,187,420	7,856,226,000	46.9	95.9	21,785,331 (83.19%)	4,353,573 (16.62%)
ZS11WL	26,777,456	8,033,236,800	47.3	96.0	22,391,549 (83.62%)	4,437,886 (16.57%)
*am*CK	27,536,936	8,261,080,800	47.1	95.9	22,589,527 (82.03%)	4,754,115 (17.26%)
*am*WL	22,117,491	6,635,247,300	46.8	96.0	18,324,147 (82.85%)	3,691,569 (16.69%)

Clean read: number of filtered sequences; clean bases: the number of sequencing sequences multiplied by the length of sequencing sequence; GC content: percentage of GC content in total base; Q30: percentage of bases with quality value ≥ 30 in the total bases; mapped reads: the number of reads mapped to the reference genome of *B. napus* and its percentage in clean reads; unique match: the number of reads mapped to the only location of the reference genome and its percentage in clean reads

**Fig. 3:**
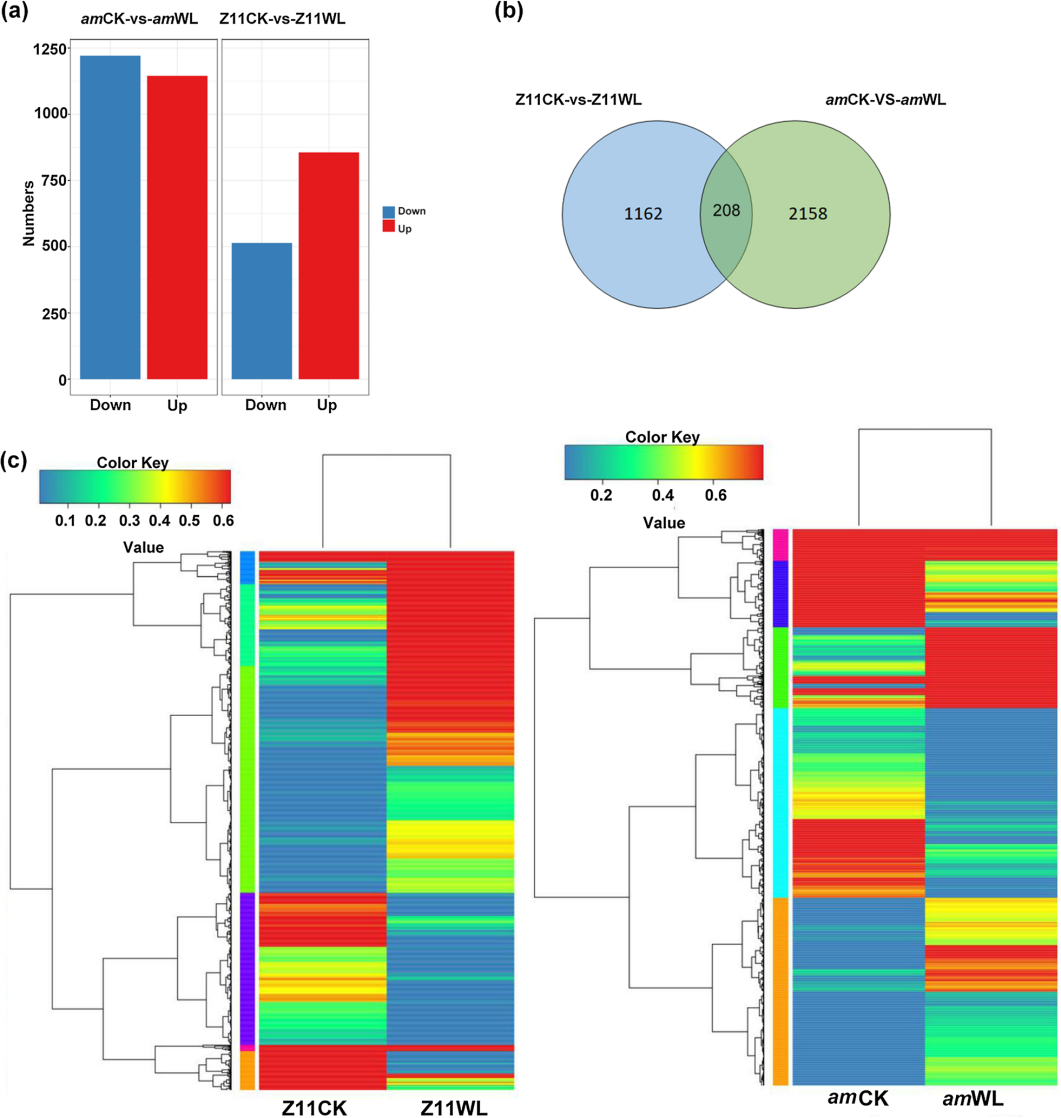
The expression profiles of waterlogging-regulated DEGs in the WT and *am* mutant. **a** Column diagram representing the numbers of DEGs in the WT and *am* mutant after the waterlogging treatment. **b** Venn diagrams showing DEGs and the overlaps of sets obtained across comparisons. **c** Heatmap clustering showing expression patterns of waterlogging-regulated DEGs based on FPKM values in the two different rapeseed cultivars

A DEG analysis was conducted to detect global expression changes in the two materials during the waterlogging process. To visually display the distributions of false discovery rate (FDR) and fold change (FC) values of all the genes between control and treatment groups of each variety, MA (minus versus add) and volcano plots of both Z11CK-vs-Z11WL and *am*CK-vs-*am*WL were constructed. As shown in Additional file 1: Fig. S5, most of the genes concentrated in the black central region and showed no significant differences in expression. The DEGs (Red dots) were more concentrated in *am*CK-vs-*am*WL, indicating that there were more genes with significantly different expression levels in the mutant. Ultimately, 1370 (856 upregulated and 514 downregulated) genes and 2,336 (1145 upregulated and 1221 downregulated) DEGs were found in the WT and mutant, respectively (Fig. 3a, Additional file 2: Tables S1, S2). Among them, there were 208 (5.95%) common DEGs in the two varieties, and the others (94.05%) were DEGS specific to only one variety. Moreover, the number of specific DEGs in the mutant was approximately twice that in the WT (Fig. 3b). To reflect the gene expression levels and patterns of multiple samples, a hierarchical cluster analysis of the DEGs in the two varieties was performed based on the fragments per kilobase of transcript per million bases (FPKM) values, which provided an overall understanding of the transcriptional changes (Fig. 3c). Using co-expression clustering, 2296 known DEGs identified in the mutant were grouped into five subclusters and 1321 known DEGs identified in the WT were grouped into six subclusters on the basis of log_10_ (FPKM + 1) values (Additional file 1: Fig. S6, Additional file 2: Tables S3, S4). These results indicated that the global gene expression profiles of the two varieties varied greatly, and there were more transcriptomic variations in the mutant than in WT after exposure to waterlogging stress.

### Functional classification and enrichment analysis of DEGs

**Fig. 4:**
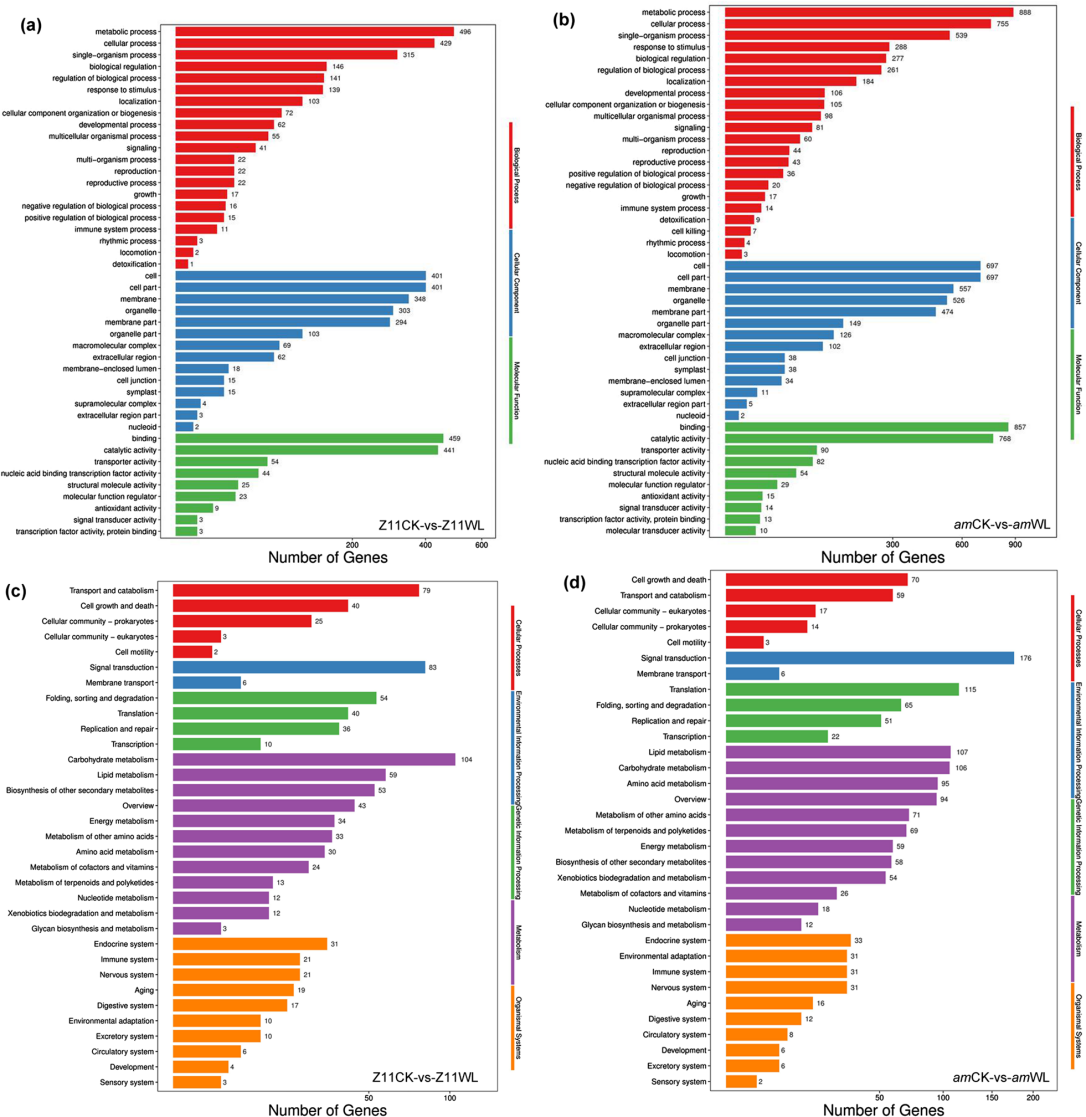
Functional classifications of DEGs based on the **a**, **b** GO annotation and **c**, **d** KEGG pathway enrichments in the WT and *am* mutant

To acknowledge the putative functions and pathways associated with the waterlogging tolerance phenotype of the *am* mutant, Gene ontology (GO) and Kyoto Encyclopedia of Genes and Genomes (KEGG) enrichment analyses of DEGs were performed. For the GO enrichment analysis, the identified DEGs annotated with GO terms were assigned to three main functional categories, cellular component, biological process and molecular function, and then were divided into 44 and 47 sub-groups in ZS11CK-vs-ZS11WL and in *am*CK-vs-*am*WL, respectively (Fig. 4a, b). Many DEGs were assigned to more than one sub-group, and the first three enriched sub-groups in each of the three main categories were the same in ZS11CK-vs-ZS11WL and *am*CK-vs-*am*WL. Most of the common DEGs in the WT ZS11 and the *am* mutant were assigned to metabolism process, binding, catalytic activity, cellular process, cell, cell part and membrane. A further analysis showed that DEGs in the mutant were more enriched in response to endogenous stimulation and hormone, oxidoreductase activity, ion channel inhibitor activity and ion channel regulator activity (Additional file 2: Table S5), whereas the WT DEGs were mainly concentrated in isocitrate lyase activity, prochlorophyllate reductase activity, peptidase regulator activity, lipid transport, 2-acylglycerol *o*-acyltransferase activity, glyoxylic acid cycle and lipid localization (Additional file 2: Table S6).

To further determine the functional distributions of these DEGs and supplemental GO classifications, we also conducted a gene functional annotation using the KEGG database. The KEGG enrichment analysis showed that the DEGs were divided into five major categories, cellular processes, environmental information processing, genetic information processing, metabolism and organismal systems. More DEGs were highlighted in ‘metabolism’ than in the other functional categories. A total of 2483 DEGs were mapped to 33 KEGG annotation pathways. As shown in Fig. 4c, the most significantly enriched pathway in WT (ZS11CK-vs-ZS11WL) was carbohydrate metabolism in the metabolic pathway branch, which contained 104 DEGs, followed by pathways related to signal transduction (83) in the environmental information processing branch and pathways related to transport and catabolism (79) in the cellular process branch (Additional file 2: Table S7). In contrast, the DEGs of *am*CK-vs-*am*WL were most significantly enriched in signal transduction (176) in the environmental information processing branch, followed by translation (115) in the genetic information processing branch and lipid metabolism (107), carbohydrate metabolism (106) and amino acid metabolism (95) in the metabolic pathway branch (Fig. 4d, Additional file 2: Table S8).

### Detection of DEGs responsible for anthocyanin biosynthesis under waterlogging-stress conditions

**Fig. 5:**
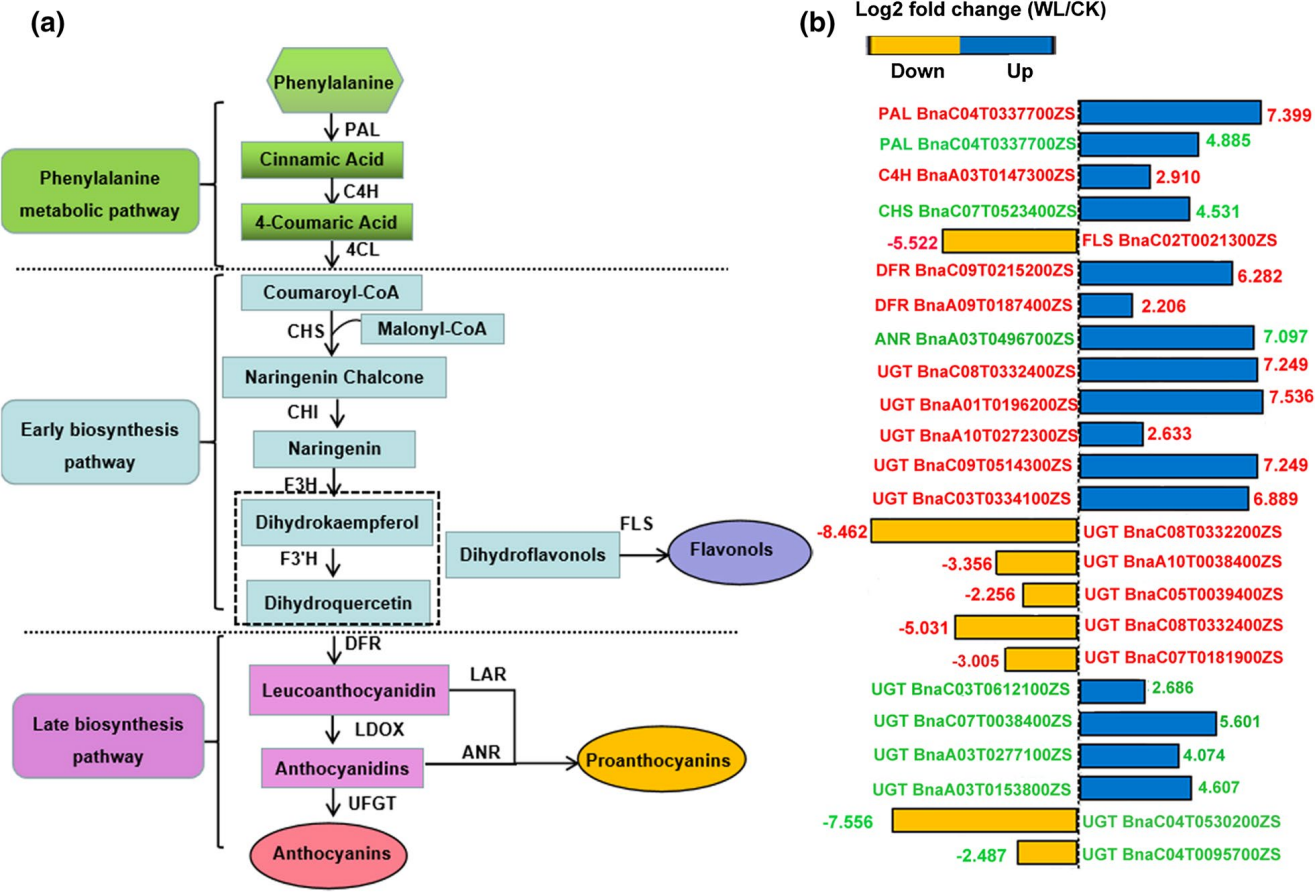
Identification of DEGs involved in anthocyanin biosynthesis in the two different rapeseed cultivars. **a** Schematic diagram of anthocyanin biosynthetic pathways and key enzymes involved in the pathways. Anthocyanin early biosynthesis-related structural genes includes genes encoding phenylalanine ammonia-lyase (PAL), cinnamate 4-hydroxylase (C4H), 4-coumaroyl:CoA ligase (4CL), chalcone synthase (CHS), chalcone isomerase (CHI), flavanone 3-hydroxylase (F3H) and flavonoid 3′-hydroxylase (F3′H). Anthocyanin late biosynthesis-related structural genes includes dihydroflavonol 4-reductase (DFR), leucoanthocyanidin dioxygenase (LDOX), UDP-flavonoid 3-*O*-glucosyltransferase (UF3GT) and anthocyanin acyltransferase (AAT). **b** Expression profiles of DEGs related to anthocyanin biosynthetic structural genes. Transcript level changes were normalized using log_2_ FC transformed counts. The blue and yellow represent up- and downregulated expression, respectively. The red and green words represent DEGs and their FCs in the mutant and WT, respectively

To elucidate the differences in waterlogging stress between the two varieties at the transcriptional level, anthocyanin biosynthetic pathways were reconstructed, and structural genes including the early and late biosynthesis genes involved in anthocyanin synthesis were indicated [37] (Fig. 5a). In this study, DEGs associated with anthocyanin production were identified and presented in Fig. 5b using a log_2_FC(WL/CK) relative transcript changes. A total of 9 genes (7 upregulated and 2 downregulated) in WT and 15 genes (9 upregulated and 6 downregulated) in the mutant were identified as anthocyanin biosynthesis-related structural genes (Fig. 5b). The expression changes in these DEGs might be related to the different colors of the two varieties under waterlogging stress. Most synthetic genes were different members of the same gene family and showed different expression patterns between the two varieties. Additionally, some family members exhibited similar expression patterns between the two varieties, but the range of the expression changes was more marked in the mutant than in the WT after waterlogging. For example, phenylalanine is a key amino acid in anthocyanin biosynthesis, and 21 DEGs involved in phenylalanine biosynthesis and related metabolic pathways, belonging to nine gene families, were identified, and most of them were upregulated in the mutant compared with in the WT (Additional file 2: Table S9). One phenylalanine ammonia-lyase (*PAL*) gene (*BnaC04T0337700ZS*) displayed similar upregulated expression profiles in the two varieties, but the increased expression level was more pronounced in the mutant than in the WT. Dihydroflavonols are common substrates of flavonol [via flavonol synthase (FLS)] and anthocyanin (via DFR) synthesis, and flavonols and anthocyanins accumulation require competition for dihydroflavonols. In our study, one *FLS* (*BnaC02T0021300ZS*) gene was significantly downregulated only in the mutant. Meanwhile, two *DFR* genes (*BnaC09T0215200ZS* and *BnaA09T0187400ZS*) displayed similar upregulation expression profiles in the mutant. Additionally, one anthocyanidin reductase (*ANR*) gene (*BnaA03T0496700ZS*) that functions in proanthocyanin production and contributes to the normal green color of plants was only upregulated in the WT. These results suggested that the *am* mutant tended to preferentially synthesize anthocyanins compared with flavonols and proanthocyanins under waterlogging-stress conditions. Leucoanthocyanidins catalyzed by DFR are further catalyzed by leucoanthocyanidin dioxygenase (LDOX) to form anthocyanidins. Although no LDOXs were detected among the DEGs, UDP-flavonoid glucosyltransferases (UFGTs), which convert anthocyanidins into anthocyanins, were significantly upregulated in the mutant. This may contribute to the increased anthocyanin content and strong waterlogging resistance in the mutant compared with the WT (Fig. 5b).

### Detection of differentially expressed transcription factors under waterlogging-stress conditions

**Fig. 6:**
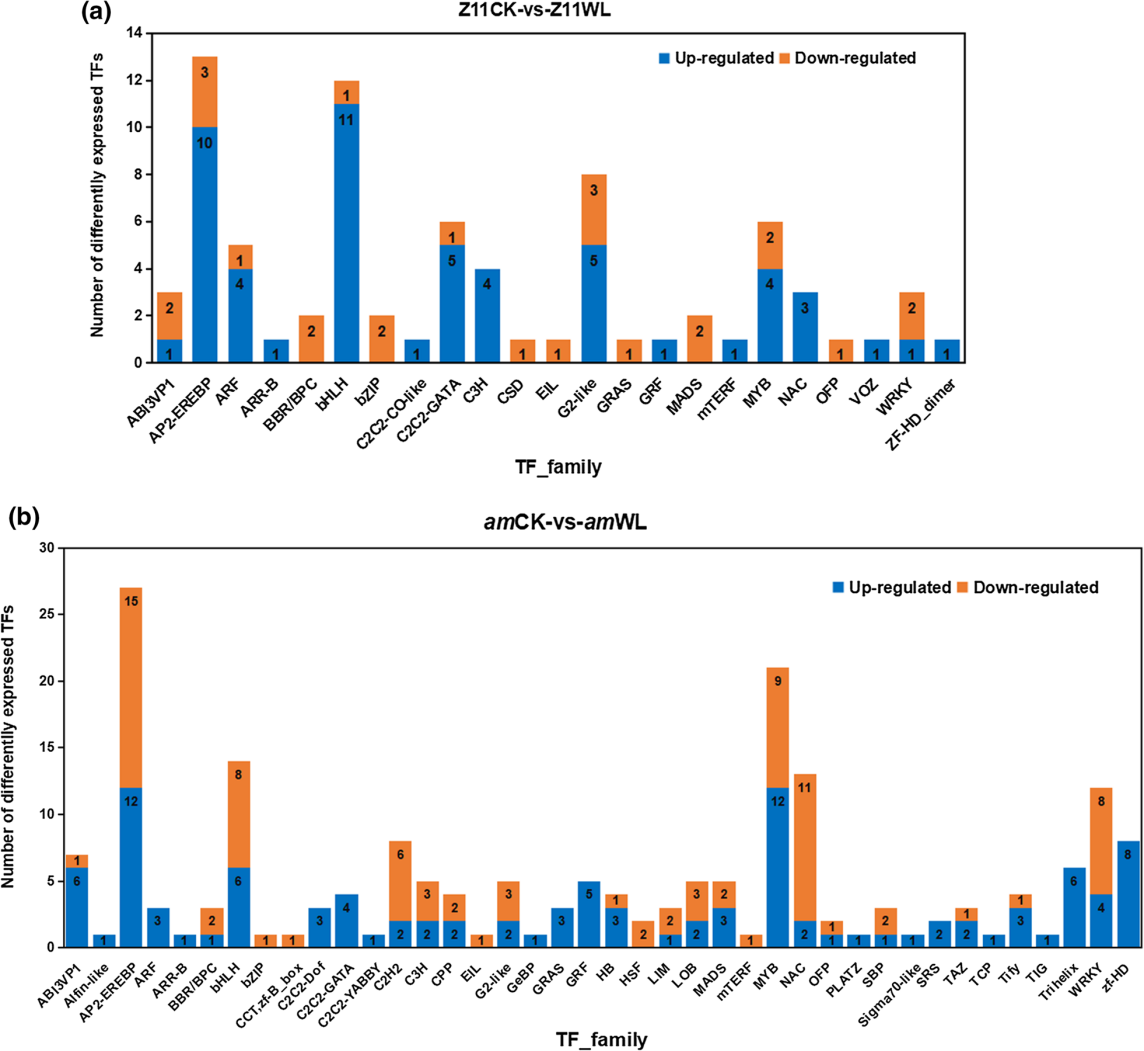
The distributions of differentially expressed TFs under waterlogging stress in **a** WT and **b** the mutant. The numbers in the histograms represent the number of up- or downregulated TFs in each TF family

The MBW complex is a well-known transcriptional regulator of anthocyanin synthesis in plants, but the other transcription factors (TFs) involved in anthocyanin synthesis are still unclear. In our study, in addition to TF members belonging to the MYB-type and bHLH families, we identified many common TF families that showed significantly changed expression levels between the WT and the mutant, such as WRKY, NAC, C2H2 and bZIP. They play an important roles in responses to biotic and abiotic stresses. Among the 3,528 DEGs, 79 genes (54 upregulated and 25 downregulated) in WT and 196 genes (110 upregulated and 86 downregulated) in the mutant were identified as TFs under waterlogging-stress conditions (Additional file 2: Tables S10, S11). They all belonged to 43 TF families (Fig. 6). In the WT, most of the upregulated TFs belonged to the bHLH (11), AP2-EREBP (10), C2C2-GATA (5) and G2-like (5) families, and the downregulated TFs mostly belonged to AP2/ERFBP (3), ABI3VP1 (2), G2-like (3), WRKY (2) and some MYB families (2) (Fig. 6a). In the *am* mutant, most of the upregulated TFs were classified into MYB (12), AP2/EREBP (12), zf-HD (8), ABI3VP1 (6) and bHLH (6) families, whereas the downregulated TFs belonged to the NAC (11), bHLH (8) and WRKY (8) families (Fig. 6b). Among them, ABI3VP1 is a family of plant-specific TFs that contains a highly conserved B3 functional domain and has a potential role in regulating seed development to germination [50]. Moreover, some TFs showed line-specific expression changes in one of the two materials, and there were more specific TFs in the mutant (52) than in the WT (3). It is noticeable that ten TF families, including Alfin-like, C2C2-Dof, C2C2-YABBY, GeBP, PLATZ, Sigma 70-like, SRS, TCP, TIG and Trihelix, showed line-specific upregulated expression changes in the mutant. These families mainly function in biological processes of plant morphogenesis, plant growth and development, photosynthesis, hormone response and stress response, whereas three TF families, one upregulated TF family VOZ and two downregulated TF families C2C2-CO-like and CSD, showed line-specific expression changes in WT. Among them, the VOZ TFs positively regulate abiotic stress tolerance [51, 52], plant defense response [53] and flowering [54, 55], and they negatively regulate PhyB-mediated seed germination in *Arabidopsis* [56].

### Detection of DEGs associated with plant hormone signal transduction

**Fig. 7:**
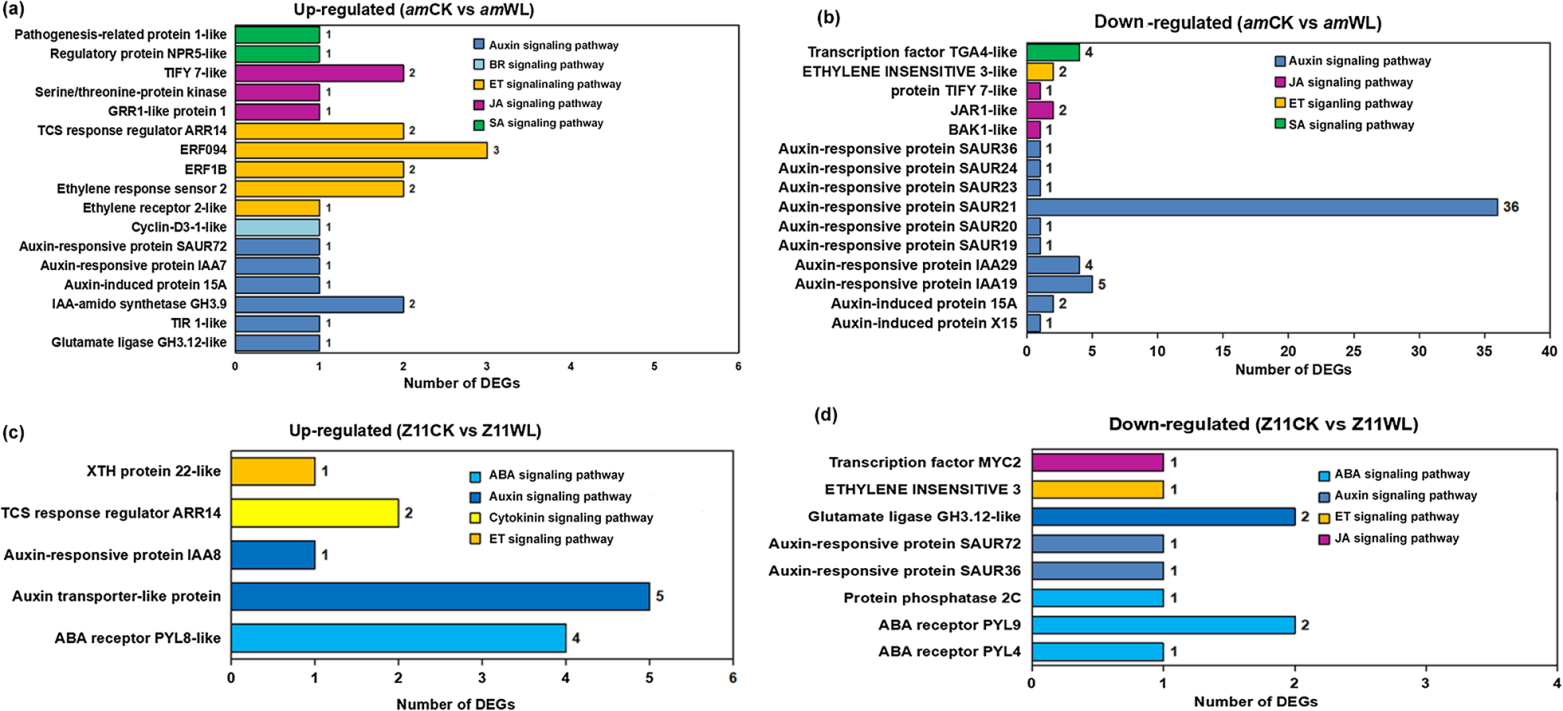
The distributions of DEGs related to plant hormone signal transduction pathways under waterlogging-stress conditions in **a**, **b** the mutant and **c**, **d** WT

Plant endogenous hormones, such as auxin, cytokinin, ABA, jasmonic acid (JA) and BR are not only growth regulators in higher plants, but they also participate in a wide range of physiological functions, such as plant responses to stress and anthocyanin synthesis [57]. There were 87 genes enriched in the term of “plant hormone signal transduction” in the mutant, including 24 upregulated DEGs and 63 downregulated DEGs (Fig. 7, Additional file 2: Table S12). Most of the upregulated DEGs belonged to the ET and auxin signaling pathways. Among them, ten DEGs involved in ET regulation, mainly including ET-responsive TFs (ERFs) and ET-response sensors, were upregulated in the mutant. A total of seven auxin signaling-related DEGs, which encoded three key proteins, auxin-responsive proteins, indole-3-acetic acid-amido synthetases and TRANSPORT INHIBITOR RESPONSE 1, were also upregulated in the mutant. Therefore, these two pathways might play important roles in anthocyanin accumulation and *B. napus* responses to waterlogging stress. Moreover, JA (3), SA (2) and BR (1) pathway-related genes were also identified among the upregulated DEGs in the mutant (Fig. 7a). Most downregulated genes in the mutant are homologs of auxin-responsive proteins SAUR (small auxin upregulated RNA) (41) and IAA (indole-3-acetic acid) (9), and auxin-induced proteins (3) (Fig. 7b). Only 23 DEGs in the WT belonged to “plant signal transduction”, including 13 upregulated DEGs and ten downregulated DEGs (Additional file 2: Table S12). Unlike in the mutant, the upregulated DEGs in the WT mainly encoded ABA receptor (4) and auxin transporter (5) homologs (Fig. 7c). Auxin (4), ABA (4), JA (1) and ET (1) signaling pathway-related DEGs were downregulated in the WT (Fig. 7d).

### Quantitative real-time PCR (qRT-PCR) validation of DEG expression levels

**Fig. 8:**
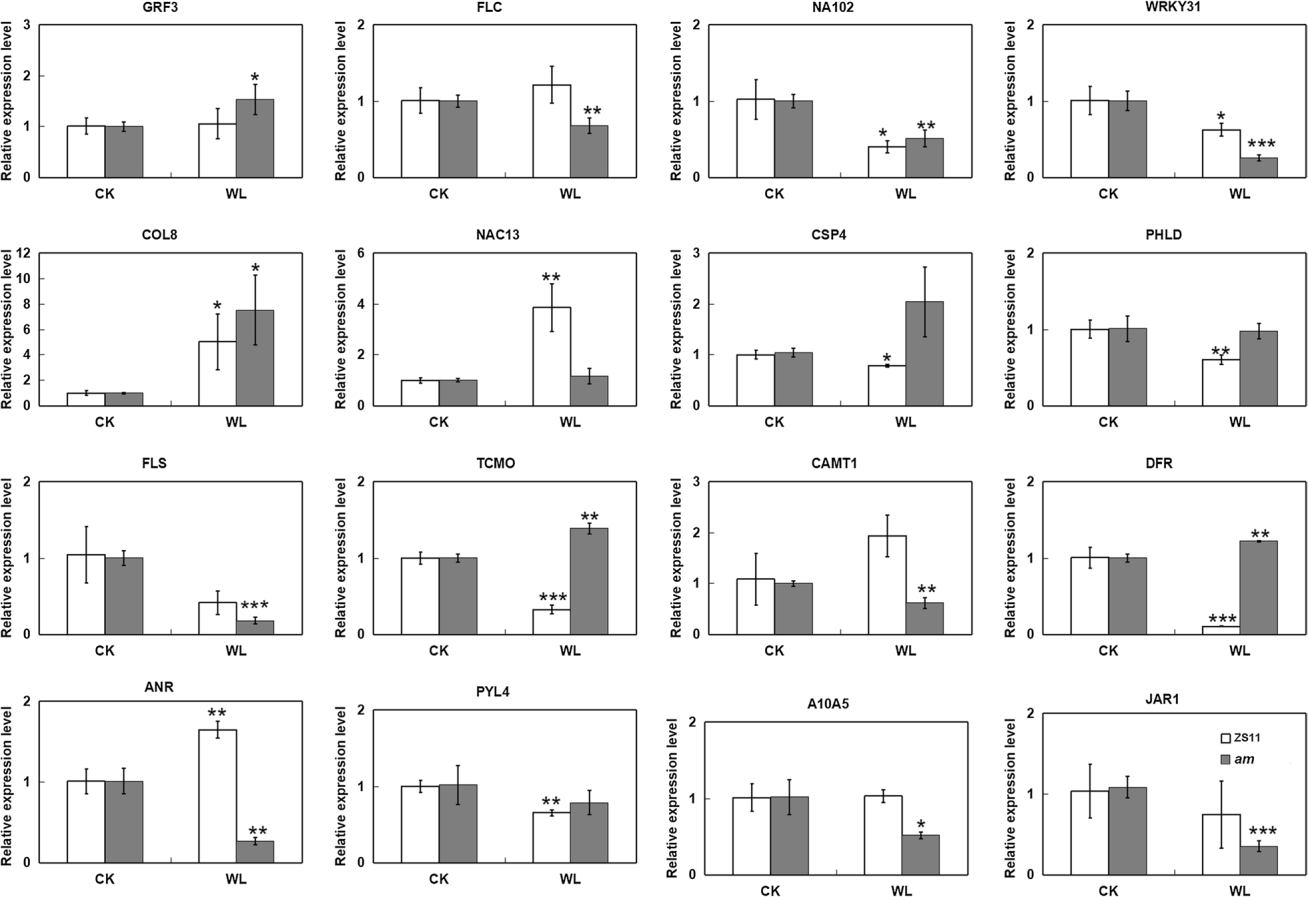
The qRT-PCR analysis of selected DEGs in *B. napus* leaves of the two different rapeseed cultivars during the waterlogging treatment. Data are presented as means ± SDs from three independent experiments. Student’s *t*-test was used for the statistical analysis between treated and untreated (control) samples in each rapeseed cultivar (*P < 0.05; **P < 0.01; ***P < 0.001)

To confirm the differential expression levels of DEGs as assessed by RNA-seq, eight key TF genes associated with the regulation of anthocyanin accumulation, five anthocyanin biosynthetic genes and three genes related to hormone signal transduction were validated by qRT-PCR (Fig. 8). The expression levels of one TF, *GRF3* (*BnaA04T0234500ZS*), and two structural genes, *TCMO* (*BnaA03T0147300ZS*) and *DFR* (*BnaC09T0215200ZS*), were all clearly upregulated in the mutant, whereas three key TFs, *FLC* (*BnaC02T0039100ZS*), *NA102* (*BnaA02T0405700ZS*) and *WRKY31* (*BnaA01T0121300ZS*), two structural genes, *FLS* (*BnaC02T0021300ZS*) and *CAMT1* (*BnaC06T0343500ZS*), and two hormone-related genes *JAR1* (*BnaC03T0263700ZS*) and *A10A5* (*BnaA01T0002400ZS*), were all significantly downregulated in the mutant. The expression patterns of these genes were in good agreement with the RNA-seq trends. The remaining six genes, *COL8* (*BnaC05T0309900ZS*), *NAC13* (*BnaC09T0521700ZS*), *CSP4* (*BnaA09T0601800ZS*), *PHLD* (*Bnascaffold0163T0000200ZS*), *ANR* (*BnaA03T0496700ZS*) and *PYL4* (*BnaA04T0246400ZS*) were differentially expressed in the WT, and also showed the same trends as the transcriptome analysis. Therefore, the qRT-PCR results were consistent with the RNA-seq data, suggesting the reliability of the transcriptome sequencing.

## Discussion

### Waterlogging stress affects phenotypic and physiological changes in the *am* mutant

As important water-soluble natural pigments in plants, anthocyanins not only play key roles in plant pollination and seed transmission, but also function in resisting abiotic and biotic stresses. There are some natural mutants that allow plants without anthocyanins to have the ability to synthesize and accumulate anthocyanins. Studying these mutants can increase our understanding of the regulatory mechanisms related to anthocyanin synthesis and metabolism. Waterlogging stress affects rapeseed germination, emergence and seedling growth, causing not only phenotypic changes, but also affecting the physiological and metabolic activities. In this study, the relative seedling survival rate and root lengths of the *am* mutant were both greater than those of WT at different times after waterlogging stress (12, 24 and 36 h) (Fig. 1). Moreover, the MDA and soluble sugar contents increased in the WT and mutant after waterlogging stress, whereas significant increases/change ranges in the mutant were observed compared with the WT (Fig. 2). MDA can reflect the degree of membrane damage caused by stress, and the accumulations of osmoregulatory substances, such as soluble sugars, can improve plant tolerance to stress by increasing osmotic potential and maintaining membrane stability [4, 58]. Our results showed significant increases in the MDA and soluble sugar contents of the *am* mutant, which were in accordance with previous studies concerning waterlogging-resistance in waterlogging-tolerant species, such as *Medicago sativa* [59], *Zea mays* L. [10] and *Arachis hypogaea* L. [60]. Thus, the mutants may reduce the damage caused by waterlogging stress by regulating the anthocyanins, MDA and osmotic adjustment substance contents. Phenotypic changes were mainly reflected by the leaves, stems and petioles of the *am* mutant changing to purple earlier than in the WT (Fig. 1c).

### Comparative transcriptome analysis of the two *B. napus* lines using RNA-seq

Because the waterlogging tolerance of rapeseed is controlled by multiple genes, omics technologies can be used to more comprehensively explore waterlogging tolerance-related proteins/genes and analyze the molecular mechanisms of waterlogging sensitivity. For example, a comparative proteomic approach revealed that the response proteins are mainly enriched in the process of redox processes, protein phosphorylation, signal transduction and metabolism, and the gene expression changes in tolerant rapeseed cultivar occur faster than in the sensitive cultivar after exposure to waterlogging treatments [8]. Transcriptome analysis showed that gene expression profiles, representing various pathways involving multiple levels of regulation, of waterlogging-tolerant rapeseed cultivar changed in response to waterlogging stress [61]. These findings provide important clues for understanding the waterlogging-tolerance mechanisms in this important economic crop. However, to date, research in this field is quite limited, which has hindered the identification of key genes that function in waterlogging tolerance and in the molecular breeding of rapeseed having a high and stable yield.

In this study, transcriptome sequencing technology was used to analyze the response of WT ZS11 and the *am* mutant to waterlogging stress at the germination stage. Many genes related to germination and growth under waterlogging stress were identified by analyzing DEGs, and these increase our understanding of the response mechanisms of rapeseed to waterlogging stress. The GO enrichment analysis revealed that the DEGs were mainly involved in metabolic process, cellular process, single biological process, biological regulation, regulation of biological process and response to stimulation (Fig. 4a). The pathway enrichment analysis showed that the DEGs were involved in signal transduction, translation process, lipid metabolism, carbohydrate metabolism and amino acid metabolism, which were the main characteristics of waterlogging germination (Fig. 4b). The transcriptome data also showed that in the *am* mutant, the most DEGs were related to signal transduction, followed by metabolism-related genes (Fig. 4a), suggesting that the mutants were more likely to use limited resources to initiate defense responses under waterlogging-stress conditions.

### DEGs related to the anthocyanin accumulation

In *Arabidopsis*, anthocyanin biosynthesis-related structural genes are divided into early and late biosynthesis genes [37]. After waterlogging stress, the mutant was more sensitive to environmental changes, and the anthocyanin content increased by approximately fivefold, which may explain the difference in seedling color between the two lines. Moreover, the changes in seedling color and anthocyanin content were related to the differential expression of structural genes involved in anthocyanin synthesis, including the early biosynthesis genes *PAL*, *C4H*, *4CL*, *CHS* and *F3H*, as well as the late biosynthesis genes *DFR* and *UFGT* (Fig. 5b), as determined by the transcriptome data. Under natural conditions and waterlogging stress, the expression levels of these key structural genes in the *am* mutant were higher than in WT, which indicated that the anthocyanin biosynthetic pathway was involved in the responses of plants to waterlogging stress, and the mutated genes in the *am* mutant may function by affecting anthocyanin anabolic metabolism. Both dihydrokaempferol and dihydroquercetin are dihydroflavonols, which represent the intermediate products of anthocyanin synthesis and can be catalyzed by FLS to form flavonoids. There are two ways to increase anthocyanin synthesis in plants. One involves increasing the accumulation of dihydroflavonols to promote anthocyanin accumulation, and the other is to decrease the FLS activity and increase the DFR activity, thereby reducing flavonoid accumulation and increasing anthocyanin the synthesis [62, 63] (Fig. 5a). In our study, the significant downregulation of FLS and significant upregulation of DFR were detected in mutants. Therefore, the mechanism leading to different anthocyanin accumulations between the two varieties may be the latter. Similarly, some varieties prioritize flavonol biosynthesis by enhancing FLS activity and strongly reduce anthocyanin accumulation by down regulating the activities of enzymes such as DFR and UFGT [64, 65]. Moreover, UFGT can convert anthocyanidins into stable anthocyanins through glycosylation. The differential expression of UFGT in the two varieties may also be related to the high accumulation of purple anthocyanins in the mutant.

### DEGs related to the regulation of anthocyanin synthesis and accumulation

The synthesis and regulatory network of plant anthocyanins is very complex, and many biosynthetic regulators of anthocyanins have been identified. The expression of genes involved in anthocyanin biosynthetic pathway is regulated by MBW complexes, which mainly regulate the expression levels of different structural genes at the transcriptional and post transcriptional levels [66, 67]. According to the results of transcriptome analysis, members of the MYB and bHLH TF families were upregulated in the two varieties, but we did not detect any WD40-repeat proteins differentially expressed between the two varieties under waterlogging conditions. In some species, such as apple and turnip, the ternary MBW complex is not necessary for the regulation of anthocyanin synthetic genes [68, 69]. Therefore, we speculated that the MYB plus bHLH dimeric complex is enough to regulate the expression of anthocyanin synthetic genes in *B. napus.* Among the differentially expressed TF genes, some belonged to the AP2/ERF TF family, which contains four subfamilies, AP2, RAV, ERF and DREB, showed specifically upregulated expression changes in the mutant. Based on our transcriptome data, only members of the AP2 and ERF subfamilies were identified as being differentially expressed, suggesting that they play a key role in the responses of rapeseed to waterlogging stress. Many studies have demonstrated that members of the AP2 and ERF subfamilies are not only key factors in stress signaling pathway [70, 71], but are also involved in plant seed development, as well as high salt and waterlogging stress responses [72–74]. The TFs that showed line-specific expression changes in the mutant, Alfin-like gene family, C2C2-Dof, C2C2-YABBY, GeBP, LOB, PLATZ, SBP, TCP, Tify, WRKY and zf-HD, which are unique to plants, played important roles in regulating plant growth and development and adversity stress responses. ABI3VP1 is also a family of plant-specific TFs that contain a highly conserved B3 functional domain, and it has a potential role in regulating seed development to germination [50, 75]. Other common TF families, such as WRKY, C2H2, NAC, ARF and bZIP, involved in plant responses to adversity, were also identified in our study. Notably, some members of TF families, including WRKY, showed inconsistent expression trends in the same material, indicating that different family members may play different roles after waterlogging stress. The WRKY TF family is involved in plant seed germination, hormone signal transduction, and the regulation of physiological and biochemical processes under stress conditions [76, 77]. Moreover, the expression patterns of these TFs genes were validated by performing qRT-PCR, which confirmed the reliability of transcriptome sequencing (Fig. 8). These TFs can also be used as targets for future functional identification.

Additionally, plant hormones are also important factors modulating anthocyanin accumulation. Several genetic mutants have been isolated, and they are mainly used to clarify the effects of hormones on anthocyanin biosynthesis. For example, the high level of ABA in *ein2* mutants may be the reason for the high anthocyanin accumulation levels in these plants [78]. Plant hormones, such as cytokinin, ABA, JA and BR play positive regulatory roles [42, 68, 79, 80], and auxin, GA and melatonin play negative roles in the synthesis of anthocyanins [81–83]. GA signals inhibit anthocyanin biosynthesis through DELLA proteins, which mediate anthocyanin biosynthesis in plant responses to abiotic stress [82], whereas JA signals participate in anthocyanin biosynthesis regulated by DELLA and restore some anthocyanin accumulation inhibited by GA [31]. In agreement with previous research results, in addition to the GA pathway, DEGs involved in auxin ABA, BR, ET and JA signaling pathways were identified to be upregulated in the mutant, and DEGs associated with the auxin and ET pathways accounted for the greatest proportions, indicating that these two pathways played key roles in response to waterlogging stress and anthocyanin accumulation in rapeseed. In addition, we also detected DEGs encoding “pathogenesis-related protein 1” and “regulatory protein NPR5” belonging to SA signaling, which showed especially upregulated expression in the mutant. Spraying SA at the early stage of grape ripening can greatly improve the anthocyanin content in fruit [84]. However, the detailed mechanisms of SA regulation in anthocyanin accumulation are still unclear and need further study.

### Screening of new transcripts after matching reference genome

After combining the genome mapping results of all the samples and removing the redundancy, the transcriptome was assembled by using the software of cufflinks, and then, the assembled transcripts were compared with the known gene annotations using cuffcompare software to obtain the classification of a reconstructed transcriptome and predict the new transcripts. An analysis of these new transcripts showed that they were mainly related to plant anthocyanin-induced response, kinase activity, signal transduction, hypersensitivity response, defense response and energy metabolism (Additional file 2: Table S13). The production of these new transcripts is an adaptive manner of gene evolution in rapeseed. Zou et al. [85] found that 11 transcripts containing early termination codons in waterlogging-tolerant varieties have specific regulatory functions under waterlogging-stress conditions. A more in-depth analysis of DEGs and the newly generated transcripts may help determine why the reason the mutants are tolerant to waterlogging stress.

## Conclusions

A natural mutant that easily induces anthocyanin production was screened from strain ZS11 of *B. napus.* Compared with the WT, the mutant showed increased tolerance to waterlogging stress during germination. A transcriptome sequencing analysis showed that the DEGs in the mutants were mainly related to signal transduction, translation and metabolism. Among them, some key genes involved in anthocyanin accumulation and hormone signal transduction, and TF genes involved in stress were differentially expressed in mutants. In the future, we will establish a mapping population for gene localization and genetic analyses, and reduce the range of candidate genes in combination with the transcriptome data. The identification and cloning of the mutant gene will contribute to the breeding of waterlogging-tolerant varieties of rapeseed.

## Materials and methods

### Plant materials

Two *B. napus* varieties WT ZS11 and its *am* mutant, having different seedling colors, from the seed bank of the School of Life Sciences at Jiangsu University were used as plant materials. The plants were grown in a plant growth room in conditions described previously [27]. Fresh leaves were harvested after a 24-h waterlogging treatment followed by an 8-day recovery period [13, 61], and corresponding untreated seedling leaves were collected for the RNA-seq and qRT-PCR experiments. Each sample had three biological replicates.

### Waterlogging treatment and phenotypic characteristics

The WT and mutant seeds were disinfected with 0.5% sodium hypochlorite solution, washed with ddH_2_O, evenly placed in a culture dish, having a diameter of 9 cm, and covered with three layers of wet filter paper. They were then germinated in an incubator for 36 h at 22 °C and 70% relative humidity with a 16-h light time. The germinated seeds (radicle length approximately 2–5 mm) of the treatment group were placed in a 10-mL centrifuge tube and subjected to waterlogging for 12 h, 24 h and 36 h. After washing with distilled water three times, they were transferred to a medium prepared with Hoagland’s nutrient solution for germination and growth for 8 days. The control group was not treated and was cultured normally. After 8 days, the survival number and seedling rate were determined. Then, ten seedlings were randomly selected and the root lengths, stem lengths and fresh weights measured. The experiment was repeated three times. The relative seedling rate, relative root length, relative stem length, relative fresh weight and relative vigor index of each seedling in both the treatment and control groups were calculated, and the relative vigor index was used as the main judgment index.

### Physiological measurement

The WT and mutant seeds germinated in the petri dish, and then the emerged germinated seeds were transplanted into a plastic flowerpot containing nutrient soil to continue growing. When the plants grew to the six-leaf one-heart stage, WT and mutant seedlings exhibiting consistent growth were selected for waterlogging treatments. The flowerpots for planting rapeseed plants were placed in a plastic box containing water to keep the water surface in the plastic box from overflowing the soil layer, and the control group was provided a normal water supply. After waterlogging for 12 days, the leaves were harvested and the related physiological indexes were measured. The chlorophyll content was determined using ethanol extraction colorimetry as described previously [48]. Under different pH conditions, anthocyanins present different colors, and the color depth is proportional to the anthocyanin content. Consequently, the anthocyanin content was determined using colorimetry in accordance with Cao et al. [86]. The MDA contents were determined using the thiobarbituric acid method at 532 nm [87]. The soluble sugar content was determined using the sulfuric acid anthrone colorimetric method at 624 nm [88]. The Coomassie Brilliant Blue method was used to quantify the soluble protein contents in plant crude extracts. The experiment was repeated three times.

### RNA extraction, library preparation, RNA sequencing and data analyses

The normally germinated WT and mutant seeds were divided into two groups, respectively. The treatment groups were subjected to waterlogging for 24 h and were recovery growth for 8 days on Hoagland medium. The control group was not treated and was cultured normally. The total RNA was extracted from the young leaves tissues of WT Z11 and the *am* mutant in control and treatment groups using an RNA extraction kit (Omega, Norcross, CA, USA) following the manufacturer’s instructions. After the RNA stock solution was diluted, the quality of each RNA sample was detected using an Agilent Technologies 2100 Bioanalyzer (Agilent Technologies, Santa Clara, CA, USA). The OD_260_/_280_ of each RNA sample was between 1.9 and 2.1, indicating that the purity levels of the extracted RNA samples were high. After extraction, electrophoretic detection was performed to observe the electrophoretic band types. Equal amounts of RNA samples from three different plants per variety were pooled. The total RNA sample quality was determined by the Wuhan FISA Gene Information Co., Ltd., and then, the cDNA library was constructed and sequenced on an Illumina Hiseq X Ten platform (Illumina Inc., San Diego, CA, USA). The raw RNA-seq data were submitted to the National Center for Biotechnology Information (NCBI) Sequence Read Archive with a Bioproject ID: PRJNA821348.

The data analysis after sequencing mainly included sequencing data quality control filtering, sequencing quality evaluation, reference sequence alignment, overall RNA-seq quality evaluation, sample correlation analysis and differential expression analysis. For the original sequencing data, reads with adaptor sequences, N (uncertain base) content > 10% and low-quality base (Q ≤ 20) content > 50% were filtered to get clean data using trimmatic software (v0.33) [89]. The statistics of sequencing data showed that Q20 > 95% and Q30 > 89%, indicating that the sequencing quality was good and could be used for subsequent comparison and data analysis. After quality control, calling by Tophat2 (v2.1.1, parameter: -library-type fr-unstranded -G) [90] and bowtie2 (v2.2.2, parameter: default) [91] was used to compare the sequence of each sample to NCBI EST database of *B. napus* and ZS11 *B. napus* reference genome (http://cbi.hzau.edu.cn/cgi-bin/bnapus//blast), and then, the alignment results were statistically analyzed by using the bowtie2 function in RSEM (v1.3.0, parameter: default) [92]. The FPKM values were used to calculate the number of aligned reads and normalize the expression levels of genes and transcripts. EdgeR (v3.6.8) package method [93] was used for screening DEGs using the thresholds of false discovery rate ≤ 0.05 and log_2_FC (fold change (WL / CK) for a gene) > 1 or log_2_FC < − 1 between untreated or waterlogging-treated groups in each variety.

### Functional and enrichment analysis of DEGs

The functions of DEGs were identified using GO (http://www.geneontology.org/) and KEGG (http://www.genome.jp/kegg/) databases. The GO enrichment was performed using GOseq, and DEGs with a corrected *p* value ≤ 0.05 were considered as significantly enriched GO terms [94]. The KEGG pathway enrichment analysis was perform using KOBAS3.0, and pathways with a corrected *p* value ≤ 0.05 were defined as significantly enriched pathways [95].

### Quantitative real-time PCR

Sub-samples (total RNA of 500 ng) of RNA-seq were treated with gDNA wiper Mix to remove genomic DNA, and then, cDNA was synthesized using Hiscript®II QRT SuperMix Kit (Vazyme, Nanjing, China) in accordance with the manufacturer’s protocols. The qRT-PCR was conducted using AceQ®qPCR SYBR®Green Master Mix (Vazyme, Nanjing, China) in accordance with the manufacturer’s instructions and run on an ABI 7300 Real Time PCR System (Applied Biosystems, Carlsbad, CA, USA). The PCR program was as follows: 95 °C for 5 min and 40 cycles of 95 °C for 10 s, followed by 60 °C (± 5 °C) for 31 s. Primer 5.0 was used to design primers, the specificity of the primer sequences was tested using a Primer-BLAST of the NCBI database, and the reasonably designed primer sequences were sent to Sangong Bioengineering (Shanghai, China) Co., Ltd. for synthesis and purification (Additional file 2: Table S14). The ACTIN gene was used as the internal control. The application of the 2^[–ΔΔC(t)]^ method converts the threshold cycle value output by the instrument into the relative gene expression level [96]. Each reaction included two biological replications with three technical replications.

## Supplementary Information

**Additional file 1: Figure S1.** The phenotypes of seedlings after 8 day of recovery growth following waterlogging for 0, 12, 24 and 36 h. Bars = 1 cm. **Figure S2.** Sequencing randomness analysis of the waterlogging-treatment and control groups in both the WT and *am* mutant. **Figure S3.** Schematic diagram of the expression level saturation distribution curve. **Figure S4.** Schematic diagram of the correlations between samples. **Figure S5.** The MA and volcano plots of gene expression in (a) *am*CK-vs-*am*WL and (b) Z11CK-vs-Z11WL. “Z11CK-vs-Z11WL” indicates waterlogging-treated WT compared with the untreated WT, and “*am*CK-vs-*am*WL” indicates waterlogging-treated *am* mutant compared with the untreated *am* mutant. **Figure S6.** Co-expression clustering showing the expression profile of DEGs in WT (a) and *am* mutant (b). The X-axis represents with or without waterlogging treatment. The Y-axis represents the value of the relative expression level [log_10_ (FPKM + 1)].

**Additional file 2: Table S1.** List of DEGs detected in the WT ZS11after the waterlogging treatment. **Table S2.** List of DEGs detected in the *am* mutant after the waterlogging treatment. **Table S3.** Differential expression of subclusters 1–5 in the *am* mutant. **Table S4.** Differential expression of subclusters 1–6 in the WT. **Table S5.** All the up- and downregulated DEGs with GO annotations in the *am* mutant. **Table S6.** All the up- and downregulated DEGs with GO annotations in the WT. **Table S7.** KEGG pathway enrichment of the DEGs in the WT. **Table S8.** KEGG pathway enrichment of the DEGs in the *am* mutant. **Table S9.** The DEG enrichment in the phenylalanine biosynthetic and metabolic pathways. **Table S10.** A list of differentially expressed TFs in the WT. **Table S11.** A list of differentially expressed TFs in the *am* mutant. **Table S12.** The DEG enrichment in plant hormone signal transduction pathways. **Table S13.** New transcript predictions as assessed by RNA-seq. **Table S14.** Primers used for qPCR validation of the selected DEGs.

## Data Availability

All data generated or analyzed during this study are included in the manuscript and additional files.

## Abbreviations

DEGs: Differently expressed genes
TF: Transcription factor
WT: Wild type
ABA: Abscisic acid
ET: Ethylene
BR: Brassinolide
CHS: Chalcone synthase
DFR: Dihydroflavonol 4-reductase
ANS: Anthocyanidin synthase
bHLH: Basic helix–loop–helix
MDA: Malondialdehyde
FDR: False discovery rate
FC: Fold change
FPKM: Fragments per kilobase of transcript per million bases
GO: Gene ontology
KEGG: Kyoto Encyclopedia of Genes and Genomes
PAL: Phenylalanine ammonia-lyase
C4H: Cinnamate 4-hydroxylase
4CL: 4-Coumaroyl:CoA ligase
CHI: Chalcone isomerase
F3H: Flavanone 3-hydroxylase
F3′H: Flavonoid 3′-hydroxylase
LDOX: Leucoanthocyanidin dioxygenase
UF3GT: UDP-flavonoid 3-*O*-glucosyltransferase
AAT: Anthocyanin acyltransferase
qRT-PCR: Quantitative real-time PCR
