# Supplementary figures and images for: Physiological and comparative transcriptome analyses reveal the mechanisms underlying waterlogging tolerance in a rapeseed anthocyanin-more mutant
Source: Biotechnol Biofuels Bioprod. 2022 May 20;15:55. doi: 10.1186/s13068-022-02155-5 (PMC9123723; doi:10.1186/s13068-022-02155-5)

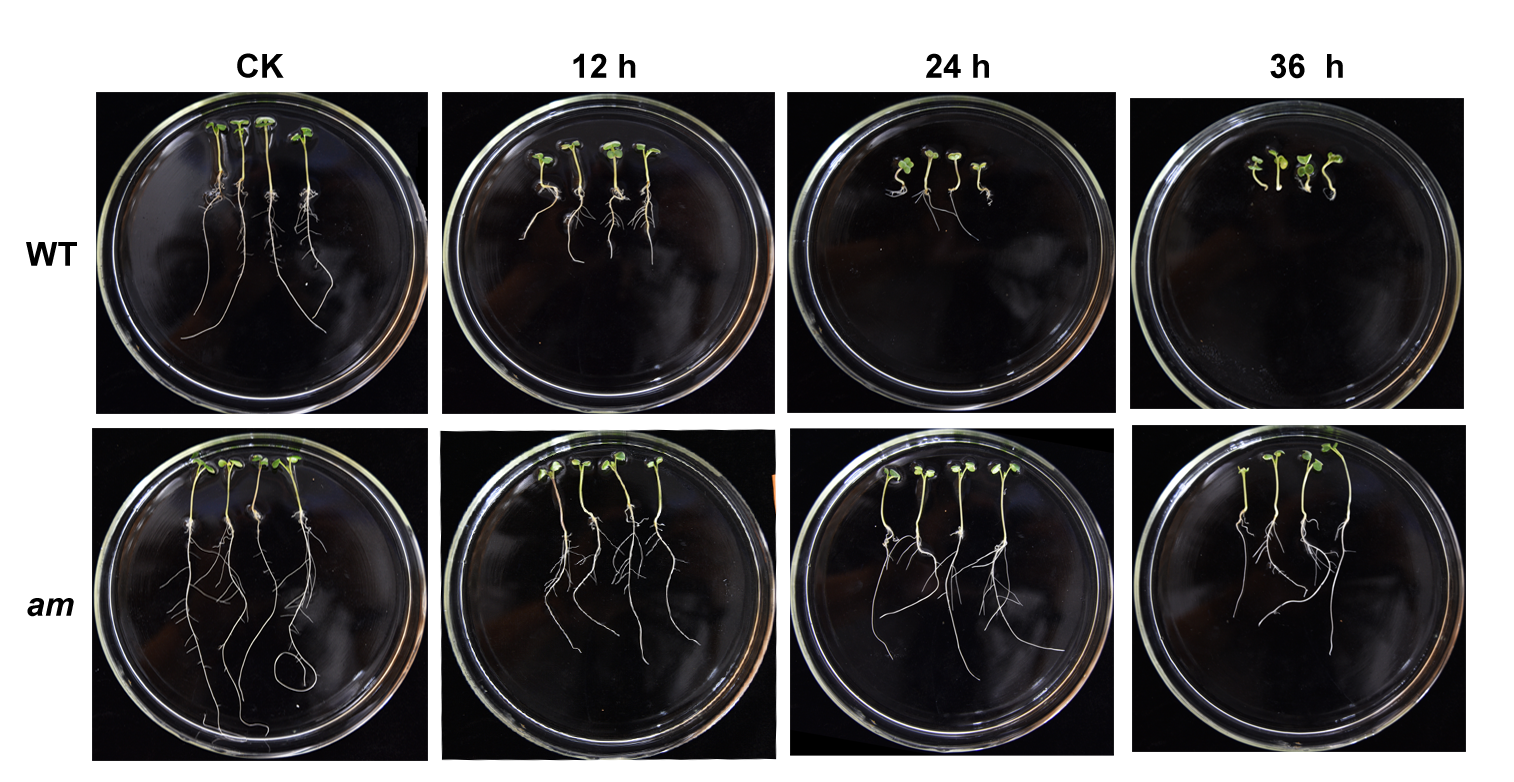

Supplement: Supplementary file 1 — Additional file 1: Figure S1. The phenotypes of seedlings after 8 day of recovery growth following waterlogging for 0, 12, 24 and 36 h. Bars = 1 cm. Figure S2. Sequencing randomness analysis of the waterlogging-treatment and control groups in both the WT and am mutant. Figure S3. Schematic diagram of the expression level saturation distribution curve. Figure S4. Schematic diagram of the correlations between samples. Figure S5. The MA and volcano plots of gene expression in (a) amCK-vs-amWL and (b) Z11CK-vs-Z11WL. “Z11CK-vs-Z11WL” indicates waterlogging-treated WT compared with the untreated WT, and “amCK-vs-amWL” indicates waterlogging-treated am mutant compared with the untreated am mutant. Figure S6. Co-expression clustering showing the expression profile of DEGs in WT (a) and am mutant (b). The X-axis represents with or without waterlogging treatment. The Y-axis represents the value of the relative expression level [log10 (FPKM + 1)]. [file 13068_2022_2155_MOESM1_ESM.zip › Fig.S1.tif]

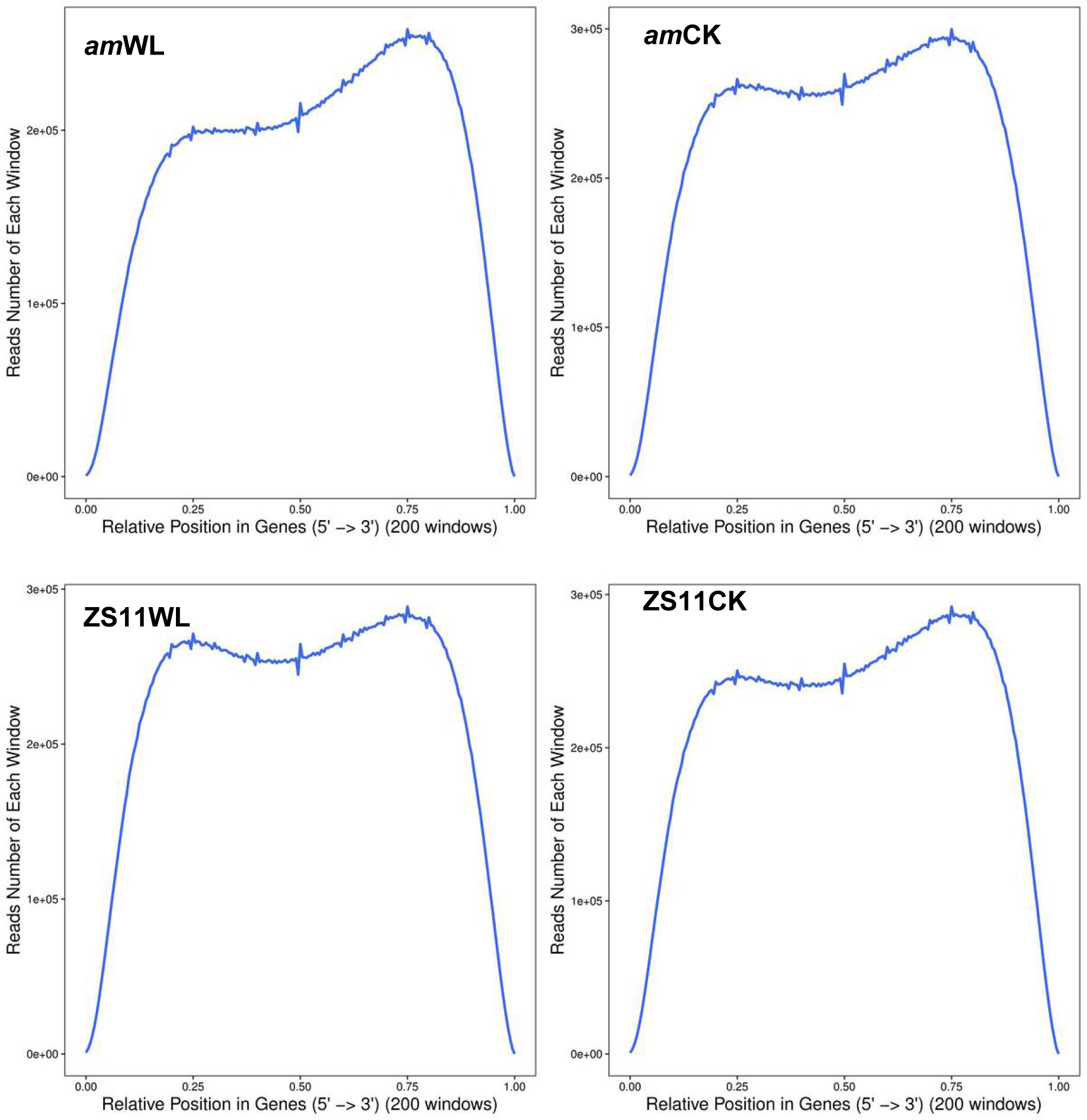

Supplement: Supplementary file 1 — Additional file 1: Figure S1. The phenotypes of seedlings after 8 day of recovery growth following waterlogging for 0, 12, 24 and 36 h. Bars = 1 cm. Figure S2. Sequencing randomness analysis of the waterlogging-treatment and control groups in both the WT and am mutant. Figure S3. Schematic diagram of the expression level saturation distribution curve. Figure S4. Schematic diagram of the correlations between samples. Figure S5. The MA and volcano plots of gene expression in (a) amCK-vs-amWL and (b) Z11CK-vs-Z11WL. “Z11CK-vs-Z11WL” indicates waterlogging-treated WT compared with the untreated WT, and “amCK-vs-amWL” indicates waterlogging-treated am mutant compared with the untreated am mutant. Figure S6. Co-expression clustering showing the expression profile of DEGs in WT (a) and am mutant (b). The X-axis represents with or without waterlogging treatment. The Y-axis represents the value of the relative expression level [log10 (FPKM + 1)]. [file 13068_2022_2155_MOESM1_ESM.zip › Fig.S2.tif]

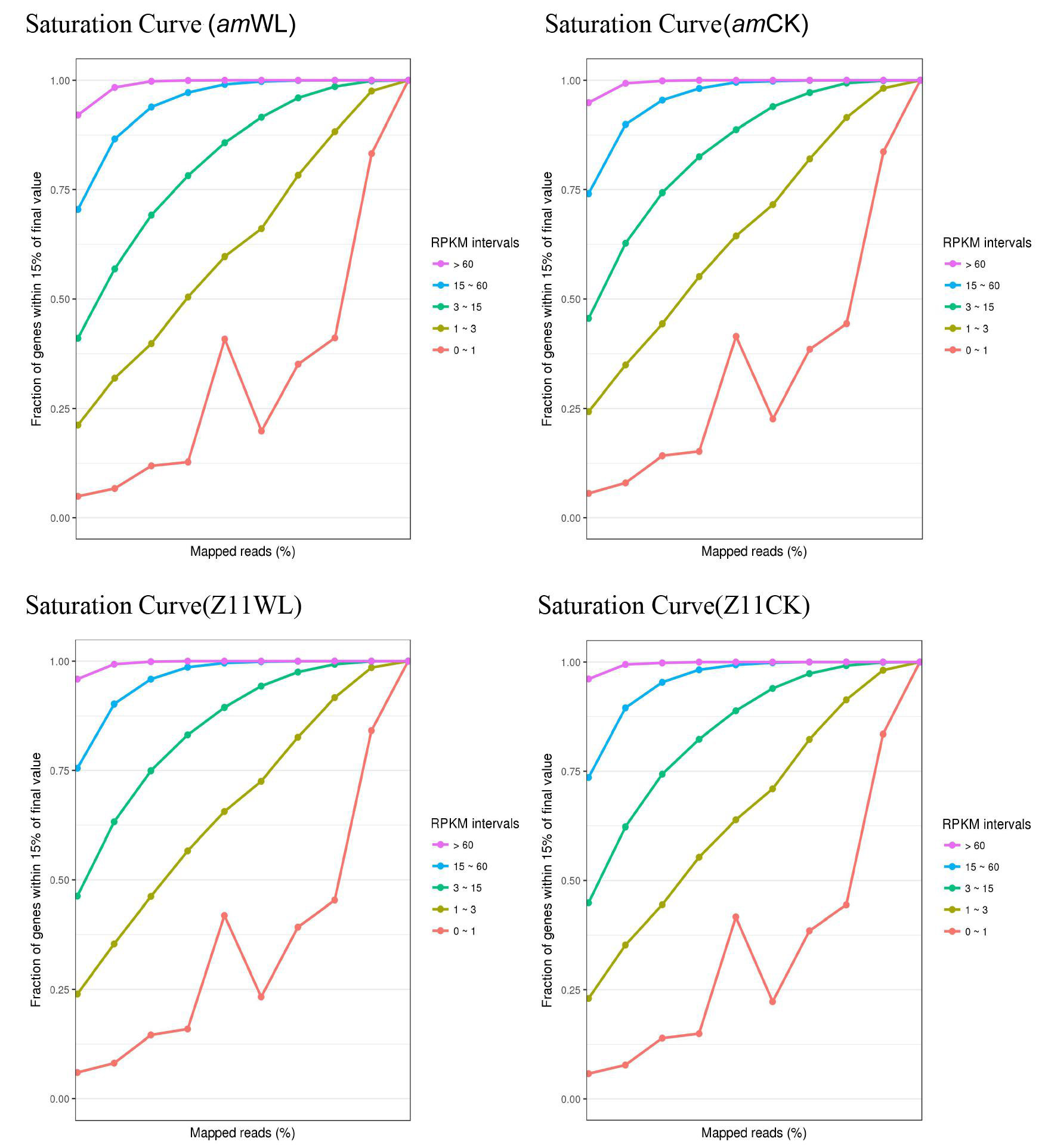

Supplement: Supplementary file 1 — Additional file 1: Figure S1. The phenotypes of seedlings after 8 day of recovery growth following waterlogging for 0, 12, 24 and 36 h. Bars = 1 cm. Figure S2. Sequencing randomness analysis of the waterlogging-treatment and control groups in both the WT and am mutant. Figure S3. Schematic diagram of the expression level saturation distribution curve. Figure S4. Schematic diagram of the correlations between samples. Figure S5. The MA and volcano plots of gene expression in (a) amCK-vs-amWL and (b) Z11CK-vs-Z11WL. “Z11CK-vs-Z11WL” indicates waterlogging-treated WT compared with the untreated WT, and “amCK-vs-amWL” indicates waterlogging-treated am mutant compared with the untreated am mutant. Figure S6. Co-expression clustering showing the expression profile of DEGs in WT (a) and am mutant (b). The X-axis represents with or without waterlogging treatment. The Y-axis represents the value of the relative expression level [log10 (FPKM + 1)]. [file 13068_2022_2155_MOESM1_ESM.zip › Fig.S3.tif]

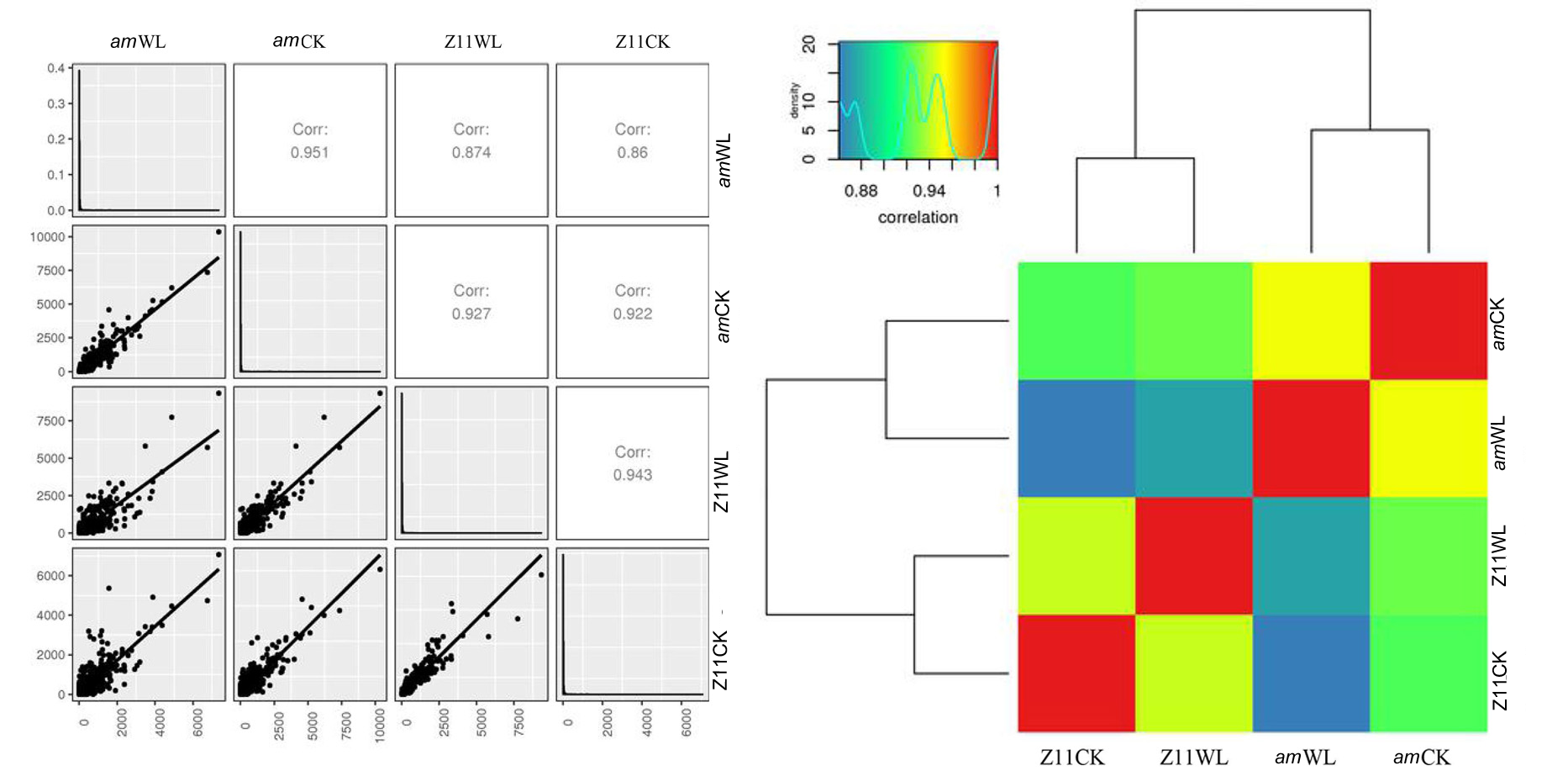

Supplement: Supplementary file 1 — Additional file 1: Figure S1. The phenotypes of seedlings after 8 day of recovery growth following waterlogging for 0, 12, 24 and 36 h. Bars = 1 cm. Figure S2. Sequencing randomness analysis of the waterlogging-treatment and control groups in both the WT and am mutant. Figure S3. Schematic diagram of the expression level saturation distribution curve. Figure S4. Schematic diagram of the correlations between samples. Figure S5. The MA and volcano plots of gene expression in (a) amCK-vs-amWL and (b) Z11CK-vs-Z11WL. “Z11CK-vs-Z11WL” indicates waterlogging-treated WT compared with the untreated WT, and “amCK-vs-amWL” indicates waterlogging-treated am mutant compared with the untreated am mutant. Figure S6. Co-expression clustering showing the expression profile of DEGs in WT (a) and am mutant (b). The X-axis represents with or without waterlogging treatment. The Y-axis represents the value of the relative expression level [log10 (FPKM + 1)]. [file 13068_2022_2155_MOESM1_ESM.zip › Fig.s4.tif]

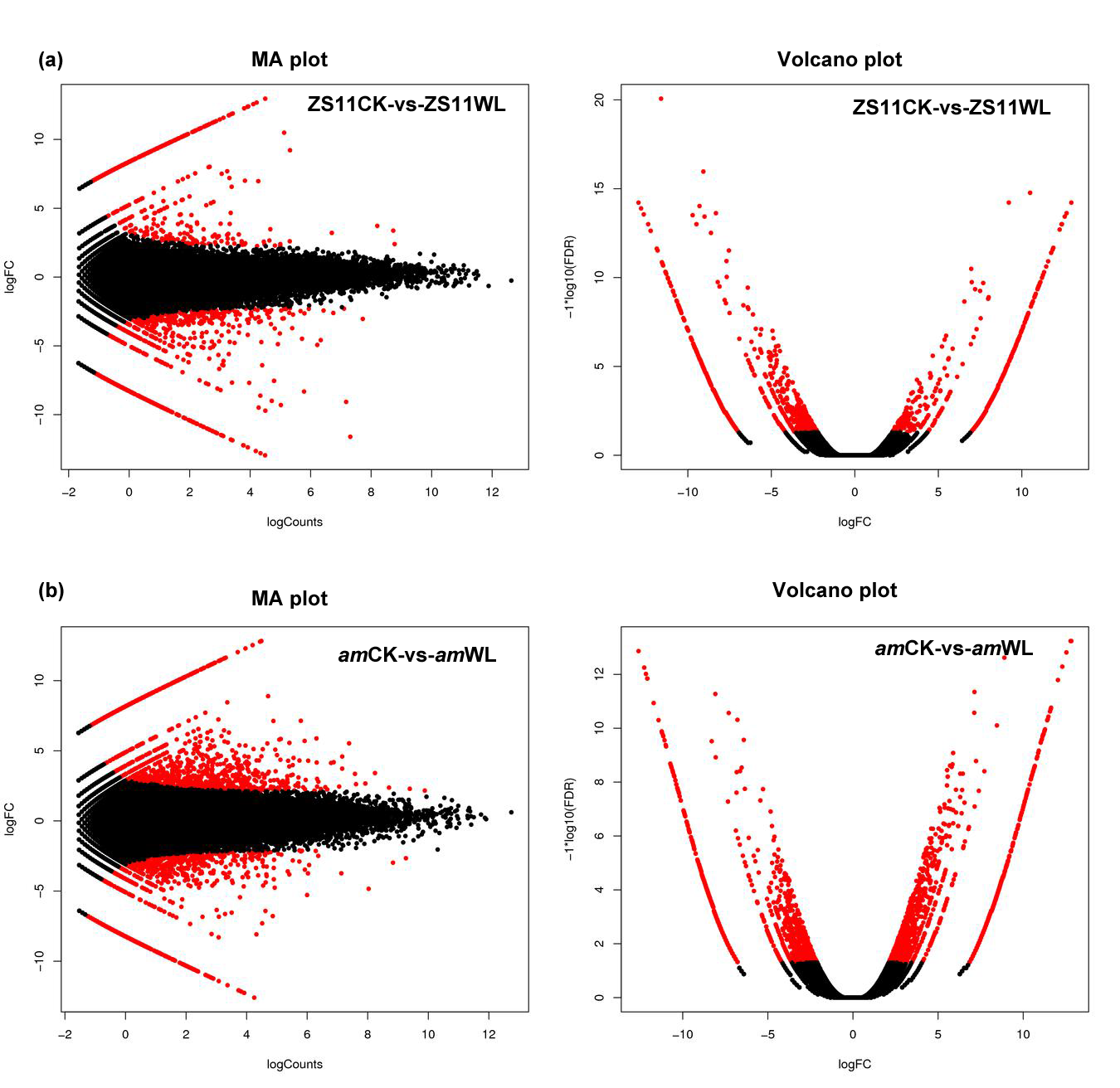

Supplement: Supplementary file 1 — Additional file 1: Figure S1. The phenotypes of seedlings after 8 day of recovery growth following waterlogging for 0, 12, 24 and 36 h. Bars = 1 cm. Figure S2. Sequencing randomness analysis of the waterlogging-treatment and control groups in both the WT and am mutant. Figure S3. Schematic diagram of the expression level saturation distribution curve. Figure S4. Schematic diagram of the correlations between samples. Figure S5. The MA and volcano plots of gene expression in (a) amCK-vs-amWL and (b) Z11CK-vs-Z11WL. “Z11CK-vs-Z11WL” indicates waterlogging-treated WT compared with the untreated WT, and “amCK-vs-amWL” indicates waterlogging-treated am mutant compared with the untreated am mutant. Figure S6. Co-expression clustering showing the expression profile of DEGs in WT (a) and am mutant (b). The X-axis represents with or without waterlogging treatment. The Y-axis represents the value of the relative expression level [log10 (FPKM + 1)]. [file 13068_2022_2155_MOESM1_ESM.zip › fig.s5.tif]

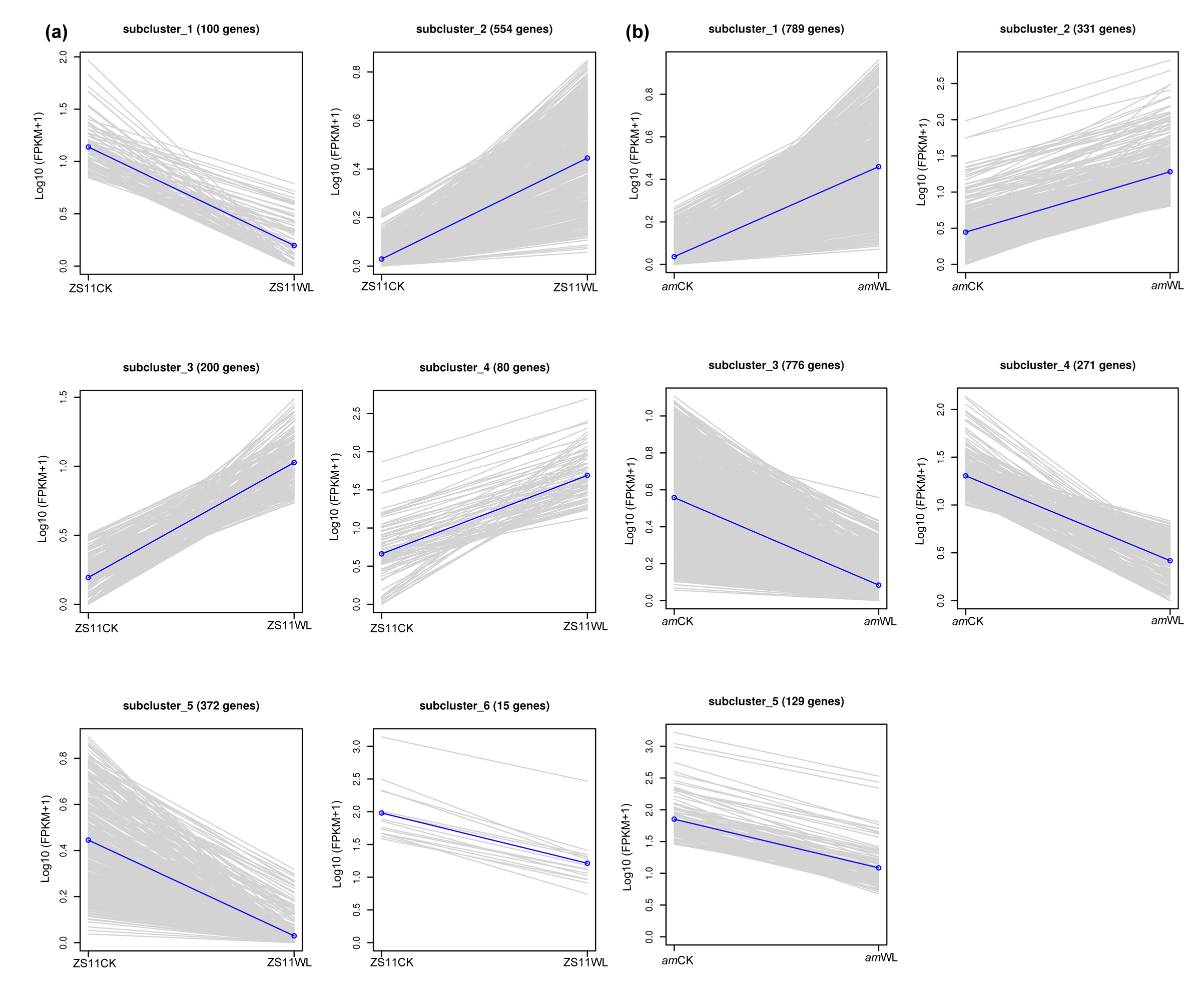

Supplement: Supplementary file 1 — Additional file 1: Figure S1. The phenotypes of seedlings after 8 day of recovery growth following waterlogging for 0, 12, 24 and 36 h. Bars = 1 cm. Figure S2. Sequencing randomness analysis of the waterlogging-treatment and control groups in both the WT and am mutant. Figure S3. Schematic diagram of the expression level saturation distribution curve. Figure S4. Schematic diagram of the correlations between samples. Figure S5. The MA and volcano plots of gene expression in (a) amCK-vs-amWL and (b) Z11CK-vs-Z11WL. “Z11CK-vs-Z11WL” indicates waterlogging-treated WT compared with the untreated WT, and “amCK-vs-amWL” indicates waterlogging-treated am mutant compared with the untreated am mutant. Figure S6. Co-expression clustering showing the expression profile of DEGs in WT (a) and am mutant (b). The X-axis represents with or without waterlogging treatment. The Y-axis represents the value of the relative expression level [log10 (FPKM + 1)]. [file 13068_2022_2155_MOESM1_ESM.zip › Fig.S6.tif]
