# Supplementary material for: Physiological and comparative transcriptome analyses reveal the mechanisms underlying waterlogging tolerance in a rapeseed anthocyanin-more mutant
Source: Biotechnol Biofuels Bioprod. 2022 May 20;15:55. doi: 10.1186/s13068-022-02155-5 (PMC9123723; doi:10.1186/s13068-022-02155-5)
Supplement: Supplementary file 2 — Additional file 2: Table S1. List of DEGs detected in the WT ZS11after the waterlogging treatment. Table S2. List of DEGs detected in the am mutant after the waterlogging treatment. Table S3. Differential expression of subclusters 1–5 in the am mutant. Table S4. Differential expression of subclusters 1–6 in the WT. Table S5. All the up- and downregulated DEGs with GO annotations in the am mutant. Table S6. All the up- and downregulated DEGs with GO annotations in the WT. Table S7. KEGG pathway enrichment of the DEGs in the WT. Table S8. KEGG pathway enrichment of the DEGs in the am mutant. Table S9. The DEG enrichment in the phenylalanine biosynthetic and metabolic pathways. Table S10. A list of differentially expressed TFs in the WT. Table S11. A list of differentially expressed TFs in the am mutant. Table S12. The DEG enrichment in plant hormone signal transduction pathways. Table S13. New transcript predictions as assessed by RNA-seq. Table S14. Primers used for qPCR validation of the selected DEGs. [file 13068_2022_2155_MOESM2_ESM.zip › Table S14.Primes.docx]

| 引物名称 | 引物序列（5’-3’） |
| --- | --- |
| COL8-F | CAGCTACTGTGTGTCTTTTCAC |
| COL8-R | ACTGATTCATCACAAACATGGC |
| NAC13-F | CAAAGGAAAAAGGCAGAGGAAA |
| NAC13-R | GTTTCATGATCTGACCCAATCG |
| PHLD-F | GTGATGTAGCTAGGCAAGAGAT |
| PHLD-R | ATTCGTTGCTTAGACGTCATTG |
| FLC-F | GATCAAATTAGGGCACAAAGGG |
| FLC-R | CTTGTCGGCTACTTTTGTTCTC |
| NA102-F | GTCTGATTTCGCCAGTATGTTC |
| NA102-R | TACAACTTTACCCCTGAGAACC |
| WRK31-F | AATGAACCCAACAAACCTCCTA |
| WRK31-R | CTGCATCAACGGGTTATTAGTG |
| GRF3-F | ACCTCCCTCATTACCAGCCT |
| GRF3-R | CGAAGACGTCCCTTGAGCAT |
| ANR-F | CAAAAGCAAGTGGATGGGTTTA |
| ANR-R | TGATGGGAGAGAAGGAGTAAGA |
| JAR1-F | GTATCATATCGTTTAGGTTCCAGAG |
| JAR1-R | AGGTTAGGTTCTGACAGAGTTG |
| CSP4-F | GCGGTTTGTAGAAGGGTAAATC |
| CSP4-R | GCAGCAACGCTTGGAATATAAT |
| FLS-F | CCACCATTAGAGAAGTGATCGA |
| FLS-R | ACGCATCTTGAAACTTTTCCTC |
| TCMO-F | GACTTTAGGTATGTGCCGTTTG |
| TCMO-R | AATCGTGATTCCCAAAATAGGC |
| CAMT1-F | AGAACCATGGAACGTATGACTT |
| CAMT1-R | ACTCCTCCAACTTTCACAAGAT |
| DFR-F | CTGCCAAGGGACGTTATATTTG |
| DFR-R | CAAACGTTGAAGGCACGTTATA |
| PYL4-F | CTCTCTTCGTTTTCAGCATACG |
| PYL4-R | CCTCGTGAGTTCAACACAAAAT |
| A10A5-F | CAGGGGCAACAAACATTCTTTA |
| A10A5-R | CAATATACCGCAGATGCAGAAG |
